# Dichotomous development of the gut microbiome in preterm infants

**DOI:** 10.1186/s40168-018-0547-8

**Published:** 2018-09-12

**Authors:** Thao T. B. Ho, Maureen W. Groer, Bradley Kane, Alyson L. Yee, Benjamin A. Torres, Jack A. Gilbert, Akhil Maheshwari

**Affiliations:** 1 Department of Pediatrics, Morsani College of Medicine, University of South Florida, Tampa, FL USA; 2 College of Nursing, University of South Florida, Tampa, FL USA; 3 Interdisciplinary Scientist Training Program, University of Chicago, Chicago, IL USA; 4 Microbiome Center, University of Chicago, Chicago, IL USA; 5 Department of Surgery, University of Chicago, Chicago, IL USA; 6 Argonne National Laboratory, Chicago, IL USA; 7 Department of Pediatrics, Johns Hopkins University, 1800 Orleans St, JHCC 8530, Baltimore, MD 21287 USA

**Keywords:** Very low birth weight infant, Gammaproteobacteria, Dysbiosis, Abbreviations, VLBWVery low birth weight, NECNecrotizing enterocolitis, OTUOperational taxonomic unit

## Abstract

**Background:**

Preterm infants are at risk of developing intestinal dysbiosis with an increased proportion of Gammaproteobacteria. In this study, we sought the clinical determinants of the relative abundance of feces-associated Gammaproteobacteria in very low birth weight (VLBW) infants. Fecal microbiome was characterized at ≤ 2 weeks and during the 3rd and 4th weeks after birth, by 16S rRNA amplicon sequencing. Maternal and infant clinical characteristics were extracted from electronic medical records. Data were analyzed by linear mixed modeling and linear regression.

**Results:**

Clinical data and fecal microbiome profiles of 45 VLBW infants (gestational age 27.9 ± 2.2 weeks; birth weight 1126 ± 208 g) were studied. Three stool samples were analyzed for each infant at mean postnatal ages of 9.9 ± 3, 20.7 ± 4.1, and 29.4 ± 4.9 days. The average relative abundance of Gammaproteobacteria was 42.5% (0–90%) at ≤ 2 weeks, 69.7% (29.9–86.9%) in the 3rd, and 75.5% (54.5–86%) in the 4th week (*p* < 0.001). Hierarchical and K-means clustering identified two distinct subgroups: cluster 1 started with comparatively low abundance that increased with time, whereas cluster 2 began with a greater abundance at ≤ 2 weeks (*p* < 0.001) that decreased over time. Both groups resembled each other by the 3rd week. Single variants of *Klebsiella* and *Staphylococcus* described variance in community structure between clusters and were shared between all infants, suggesting a common, hospital-derived source. Fecal Gammaproteobacteria was positively associated with vaginal delivery and antenatal steroids.

**Conclusions:**

We detected a dichotomy in gut microbiome assembly in preterm infants: some preterm infants started with low relative gammaproteobacterial abundance in stool that increased as a function of postnatal age, whereas others began with and maintained high abundance. Vaginal birth and antenatal steroids were identified as predictors of Gammaproteobacteria abundance in the early (≤ 2 weeks) and later (3rd and 4th weeks) stool samples, respectively. These findings are important in understanding the development of the gut microbiome in premature infants.

**Electronic supplementary material:**

The online version of this article (10.1186/s40168-018-0547-8) contains supplementary material, which is available to authorized users.

## Background

In newborn infants, the enteric microbiome is an important influence on mucosal immunity, nutrient absorption, and energy regulation in the developing intestine [1]. Healthy full-term neonates acquire a “core” enteric microbiome with the inocula received during and after birth from the maternal microbiota in the vaginal, fecal, and cutaneous compartments and from maternal milk [1, 2]. The gut microbiome in these infants shows dominance of Gram-positive Firmicutes such as Staphylococcus, Propionibacterium, Bifidobacterium, and Lactobacillus [2, 3]. However, in marked contrast to term infants, preterm infants are at risk of delayed and altered assembly of their intestinal microbiome [4, 5]. These patients have several clinical and physiological constraints, including absent or limited exposure to maternal microbiota due to shortened labor or cesarean birth, mucosal immaturity, and the lack of physical and immune defenses such as gastric acid, secretory IgA, and intestinal mucus, frequent multisystem organ dysfunction with consequent exposure to broad-spectrum antibiotics and various indwelling tubes and catheters, delays in enteral feeding, and intestinal dysmotility [3, 5–8]. Many premature infants show dysbiosis with a preponderance of Gram-negative bacteria of the class Gammaproteobacteria and its constituent families *Enterobacteriaceae*, *Vibrionaceae*, and *Pseudomonadaceae* [9–14]. There are concerns that such dysbiosis in preterm infants may be associated with adverse outcomes, including necrotizing enterocolitis, late-onset sepsis, and developmental delay [5, 10, 11, 15].

In this prospective observational study, we investigated the clinical antecedents of increased relative abundance of fecal Gammaproteobacteria in premature infants. Our goal was to identify the clinical characteristics of preterm infants who developed enteral dysbiosis, which in turn, could inform future efforts to direct microbiome screening in a clinical setting. We hypothesized that most premature infants begin with few Gammaproteobacteria in their stool and acquire these bacteria from the hospital microenvironment or from human interaction [16–19], as a function of postnatal age. We reasoned that once introduced into the relatively uninhabited preterm intestine [20], the abundance of gammaproteobacterial taxa would expand with time. Therefore, we posited that (a) dysbiosis is a stable state wherein fecal Gammaproteobacteria would either increase in relative proportion or remain stable, but not decrease over time; and (b) Gammaproteobacteria-enriched dysbiosis may be seen in a majority of convalescing premature infants. To investigate these hypotheses, we recorded the demographic and clinical information from a cohort of inborn, very low birth weight (VLBW) infants and analyzed their fecal microbiome at serial time-points during the first month after birth.

## Methods

### Demographic and clinical information

This prospective study was performed after approval by Institutional Review Boards at University of South Florida and Tampa General Hospital (TGH). We enrolled all eligible VLBW infants admitted to the neonatal intensive care unit (NICU) at TGH, an academic regional referral center with a single-patient room floor plan, during the period May 2012–December 2013. Inclusion criteria included informed parental consent and the availability of a stool sample ≤ 2 weeks after birth. Infants with major congenital anomalies were excluded. The following maternal and neonatal information was obtained from medical records: maternal Hispanic ethnicity, maternal race (Black, White, Asian, and others), maternal age, duration of ruptured membranes, clinical chorioamnionitis, maternal hypertension, diabetes, and her body mass index; antenatal treatment with steroids and magnesium sulfate; mode of delivery, gestational age, birth weight, postnatal age, postmenstrual age (gestational age at birth + postnatal age, in weeks), gender, small-for-gestational age (SGA), singleton/multiple gestation, Apgar scores, admission temperature, early-onset or any sepsis (positive blood culture), respiratory distress syndrome, surfactant use, need for supplemental oxygen and/or positive pressure, patency of the *ductus arteriosus*, postnatal treatment with steroids or indomethacin/ibuprofen, days of antibiotic treatment during the first 2 weeks and the total number of days on antibiotics during the entire hospital stay, red blood cell transfusions, feeding (exclusive maternal/donor breast milk, exclusive formula, and mixed), NEC (Bell stages II or III [21]), chronic lung disease (need for supplemental oxygen at 36 weeks’ corrected gestational age), extra-uterine growth restriction, and the length of hospital stay.

### Fecal DNA amplification

Stool samples obtained at ≤ 2 weeks, the 3rd week, and the 4th week after birth were stored at − 80°C under uniform conditions until analysis [22]. Total DNA from 100 to 250 mg stool (MoBio PowerFecal DNA kit, Qiagen, Carlsbad, CA) was used to amplify the V4 region of 16S rRNA gene using polymerase chain reaction with modified 515F and 806R primers [23, 24]. These DNA segments were sequenced using the MiSeq platform (Illumina, San Diego, CA) to generate about 15,000,250 base-pair paired-end reads per sample [24].

### Statistical analysis

Demultiplexed DNA sequences were analyzed for bacterial identification to genus level using the CLC Biomedical Workbench 3.5.3 (Qiagen) using the default setting. Operational taxonomic units (OTUs) were assigned based on 97% sequence identity to the Greengenes v13.8 reference database, and their relative abundance (percentage) was computed. Bacterial diversity was measured within samples (alpha-diversity) as the number of OTUs, phylodiversity, and the Chao 1, Simpson, and Shannon indices; between samples (beta-diversity) by principal coordinate analysis and permutational multivariate analysis of variance (PERMANOVA) of weighted and unweighted UniFrac distance matrices, Jaccard coefficient, and the Bray-Curtis dissimilarity index. To improve upon the taxonomic resolution of the microbial analysis, we characterized OTUs to single nucleotide variants (SNVs) using Divisive Amplicon De-noising Algorithm 2 (DADA2) [25]. The V4 region 16S rRNA gene amplicon data was analyzed using the DADA2 pipeline. First, the demultiplexed fastq files were filtered and trimmed. Each sample was dereplicated, a portion of the data set was used to estimate the error parameters, and dada() was applied to the full pooled data set using those inferred error parameters. Paired reads were then merged, and removeBimeraDenovo() was used to remove chimeras. Taxonomy was assigned against the Greengenes v13.8 database (see Additional file 1 for Code for DADA2). Volatility analysis was performed by comparing unweighted UniFrac distances on SNVs between subgroups. To identify the predictive value of subgroups on microbiome community composition, we applied random forest machine learning (after rarefying to 5000 sequences/sample, 1000 trees) and Analysis of Composition of Microbiomes (ANCOM) [26]. Finally, to determine whether the data on relative abundance/proportions reflect a change in the absolute abundance of specific taxa, we performed Balance Tree Analysis using Gneiss [27].

Clinical information was analyzed using SPSS (IBM, Armonk, NY). Scalar variables were compared by the Mann-Whitney *U* [28] or Student’s *t* test [29], and categorical variables by Fisher’s exact test [30]. We used the linear mixed-effects modeling procedure [31] to identify determinants of fecal bacterial colonization. The linear mixed-effects procedure was performed using the maximum likelihood method. The autoregressive covariance matrix (with heterogeneous variances) was used as the dependent variables were anticipated to diverge with time. Best-fitting models were identified for lowest values of the − 2 log likelihood, Akaike’s information criterion, and Schwarz’s Bayesian criterion [32]. Important independent variables were shortlisted using bootstrap bagging [33], where a bootstrap dataset was constructed by not sampling a third of all subjects and replacing these by an equal number of duplicated samples. The bootstrap sample was analyzed by logistic regression with entry criterion of *p* < 0.2. The number of times a risk factor appeared in these 1000 analyses was taken as a reflection of the reliability. Because of the limited number of subjects in the study cohort and concern about model overfitting, multivariable analyses were limited to biologically plausible associations, to main effects for baseline measures, and time-dependent covariates for longitudinal measures. Models were adjusted for birth weight, gestation, and postmenstrual age. To ensure stability/reliability of estimates, 95% confidence intervals (CI) were re-estimated by bootstrapping (*n* = 1000). We also performed linear regression to identify the determinants of gammaproteobacterial abundance at each time-point of stool collection. Variables identified for the mixed-effects analysis were tested with entry at *p* < 0.2 and acceptance at *p* < 0.05, first using a one-step forced entry and then “stepwise” in the sequence of appearance during perinatal period [34]. To identify highly correlated variables, multicollinearity diagnostics (tolerance values < 0.2, variance inflation factors > 10) were reviewed [35]. The independence of variables was confirmed by the Durbin-Watson statistic (models accepted if between 1.5 and 2.5) [36]. Scatterplots of standardized residuals vs. standardized predicted values were evaluated for homoscedasticity and nonlinearity. Normality of residuals was confirmed by evaluation of histograms and normal probability plots. Statistical tests were two-tailed and considered significant at *p* < 0.05.

## Results

### Demographic and clinical information

We enrolled 45 eligible VLBW infants admitted to our NICU between May 2012 and December 2013. These infants were born at a gestation (mean ± standard deviation, SD) of 27.9 ± 2.2 weeks, with birth weight 1126 ± 208 g. Their clinical characteristics are summarized in Table 1.

**Table 1:** Perinatal and neonatal clinical characteristics

Characteristic	*N* = 45
Gestational age, weeks, (mean, SD)	28 (2)
Birth weight, grams, (mean, SD)	1126 (208)
Male	21 (46.7%)
Hispanic ethnicity	9 (20%)
Race	
Black	19 (42.2%)
White	25 (55.6%)
Antenatal medications	
Steroids	39 (86.7%)
Magnesium	34 (75.6%)
Vaginal birth	11 (24.4%)
Multiple birth	7 (15.6%)
Chorioamnionitis	26 (47.8%)
Maternal hypertension	13 (28.9%)
Small for gestational age	3 (6.7%)
Respiratory distress syndrome	26 (57.8%)
Oxygen on day 28	11 (24.4%)
Oxygen on day 36	2 (4.4%)
Patent ductus arteriosus	6 (13.3%)
Indomethacin	2 (4.4%)
Patent ductus arteriosus ligation	1 (2.2%)
Treated retinopathy of prematurity	0
Necrotizing enterocolitis	1 (2.2%)
Surgical necrotizing enterocolitis	1 (2.2%)
Days on antibiotics, (mean, SD)	4.5 (4.7)
Positive blood culture	5 (11.1%)
Packed red blood cell transfusion	16 (35.6%)
Feeding type	
Maternal breast milk only	25 (55.6%)
Formula only	2 (4.4%)
Mixed feeding types	18 (40.0%)
Discharge weight < 10th percentile	11 (24.4%)
Length of stay, days, (mean, SD)	68 (29)

### Fecal microbiome

Three stool samples were analyzed from all infants, obtained at the postnatal age (mean ± SD) 9.9 ± 3, 20.7 ± 4.1, and 29.4 ± 4.9 days, respectively. The postmenstrual age at these time-points was 29.8 ± 2.3, 31.2 ± 1.9, and 32.6 ± 1.9 weeks, respectively. A total of 2,017,727 reads was obtained, with a mean 15,285 (± standard deviation 7139) sequences per sample. One of the stool samples at the first time-point had inadequate biomass for DNA sequencing and was excluded. The alpha-diversity metrics (number of OTUs, phylodiversity, and Shannon, Chao1, and Simpson indices) increased with postnatal age (see Additional file 2: Table S1).

### Major bacterial communities in stool

**Fig. 1:**
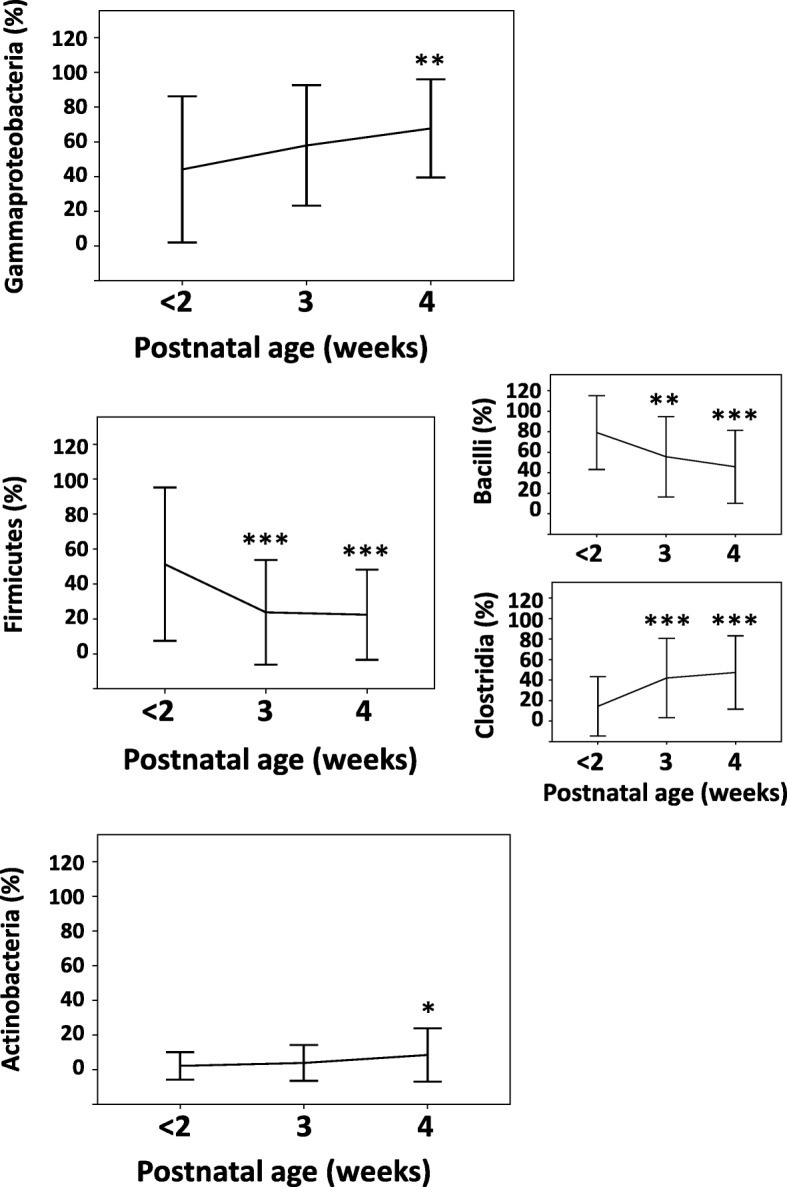
Relative abundance of major bacterial taxonomic units in stool over time. Line diagrams (means ± standard deviation) show the relative abundances of major bacterial taxonomic units in stool, by cluster. Stool samples were collected during the first 2 weeks, and then during the 3rd and the 4th weeks, respectively. Repeated measures analysis of variance; *p < 0.05, **p < 0.01, and ***p < 0.001

Proteobacteria increased in abundance over time, comprising 46% (median; interquartile range/IQR = 0–90%) of all reads at ≤ 2 weeks, 83.5% (54.8–93.3%) in the 3rd and 77% (57–88.3%) in the 4th week (*p* < 0.001). The class Gammaproteobacteria dominated the Proteobacteria, comprising 42.5% (0–90%) at ≤ 2 weeks, 69.7% (29.9–86.9%) in the 3rd, and 75.5% (54.5–86%) in the 4th week (*p* < 0.001) (Fig. 1, Additional file 2: Table S2). Gammaproteobacteria comprised > 50% reads in 20/44 (45.5%) infants at ≤ 2 weeks, 29/45 (64.4%) in the 3rd week, and 36/45 (80%) in the 4th week. *Klebsiella* were the predominant gammaproteobacterial genus, dominating nearly all infants (median 44%, range 0–100% at ≤ 2 weeks, 85% (0–99%) in the 3rd, and 78.5% (0–99%) in the 4th week (changes not significant because of high inter-infant variability). A SNV mapping to *Klebsiella* comprised 0.18% of all reads (median, range 0–99.4%) at ≤ 2 weeks, 24.6% (0–99.5%) in the 3rd (*p* = 0.034), and 26.2% (0–99.4%) in the 4th week (not significant vs. ≤ 2 week samples). Identified gammaproteobacterial genera are listed in Additional file 2: Table S3.

Firmicutes were the second most abundant phylum at ≤ 2 weeks (median 41.5%, IQR 3.25–100%). The class Bacilli accounted for nearly all Firmicutes at ≤ 2 weeks (100%, IQR 60–100%), whereas Clostridia were dominant during the 4th week (42%, IQR 8.5–85%; *p* < 0.001). At the genus level, *Staphylococcus* were abundant at ≤ 2 weeks and decreased with age, whereas *Enterococcus* increased over time. *Lactobacillus* were scant. Other Firmicute genera are presented in Additional file 2: Table S4. Actinobacteria and Bacteroidetes were nearly absent from this cohort (see Additional file 2: Table S2).

### Cluster analysis for fecal abundance of Gammaproteobacteria

**Fig. 2:**
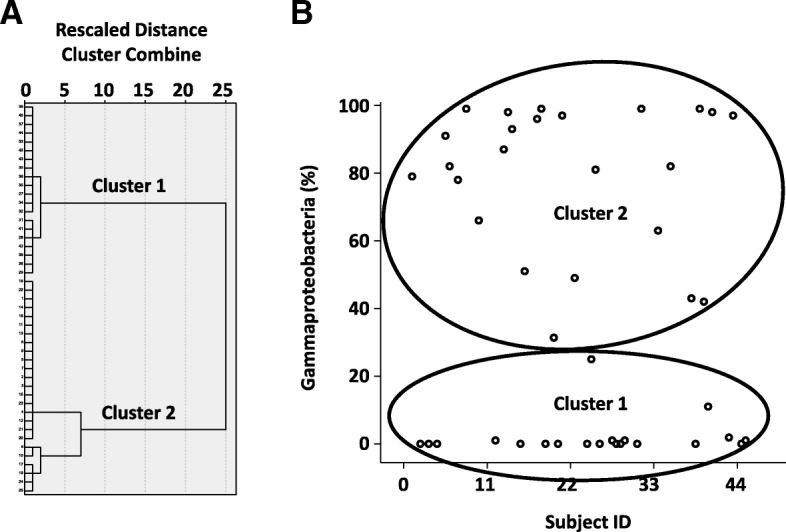
Clustering of VLBW infants by the relative abundance of fecal Gammaproteobacteria. **a** Dendrogram shows the average linkage (between the two groups) derived by hierarchical clustering. **b**. Scatter-plot shows that the VLBW infants included in our study were grouped into two distinct clusters based on the relative abundance of *Gammaproteobacteria* (percentages) in stool samples obtained during the first two postnatal weeks

The relative abundance of Gammaproteobacteria in ≤ 2 week samples varied widely (0–90%; see Additional file 2: Table S2). Therefore, we looked for evidence of clustering in our cohort. Hierarchical and K-means clustering [37] of Gammaproteobacteria percentages in the 44 first time-point stool samples showed 2 subgroups (Fig. 2a, b): cluster 1 (20 infants) started with low gammaproteobacterial abundance (mean ± SD 2.09 ± 5.91%, median 0, range 0–25%), whereas cluster 2 (24 infants) showed greater gammaproteobacterial relative abundance (mean ± SD 79.18 ± 21.6%, median 84.5%, range 31.36–99%; *p* < 0.001). Cluster 1 infants had lower birth weight (mean ± SD 1053 ± 227 g vs. 1176 ± 175 g in cluster 2; *p* = 0.049) and were less likely to have had a vaginal birth (1/20 in cluster 1 vs. 10/24 in cluster 2, *p* = 0.006)*.* Their clinical characteristics are summarized in Additional file 2: Table S5, and the OTUs in Additional file 2: Table S6 a–c. Multiple birth participants sorted to the same cluster as their siblings, indicating the validity of this grouping. Random forest analysis predicted cluster identity with a prediction/error ratio of 2.2, but did not identify any SNVs with a large feature importance score (see Additional file 2: Table S7).

**Fig. 3:**
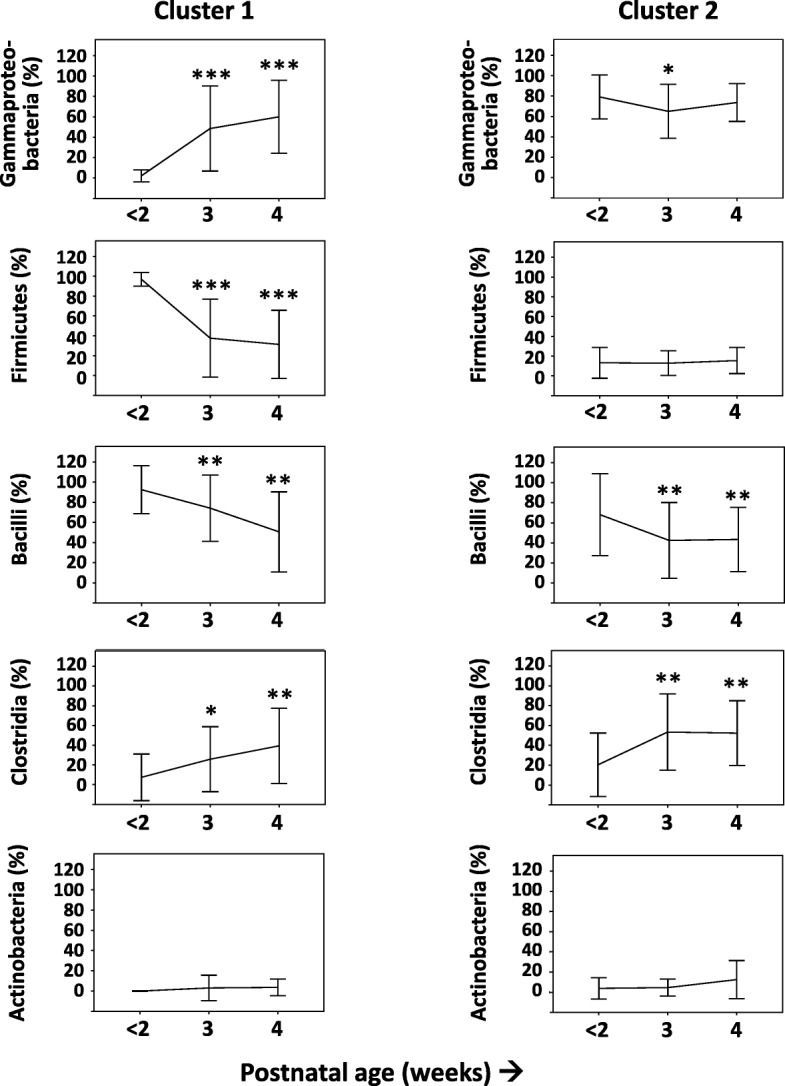
Relative abundance of major bacterial taxonomic units in stool, by cluster. Line diagrams (means ± standard deviation) show the relative abundances of major bacterial taxonomic units in stool in clusters 1 and 2. Stool samples were collected during the first 2 weeks, and then during the 3rd and the 4th weeks, respectively. Repeated measures analysis of variance; *p < 0.05, **p < 0.01, and ***p < 0.001

**Fig. 4:**
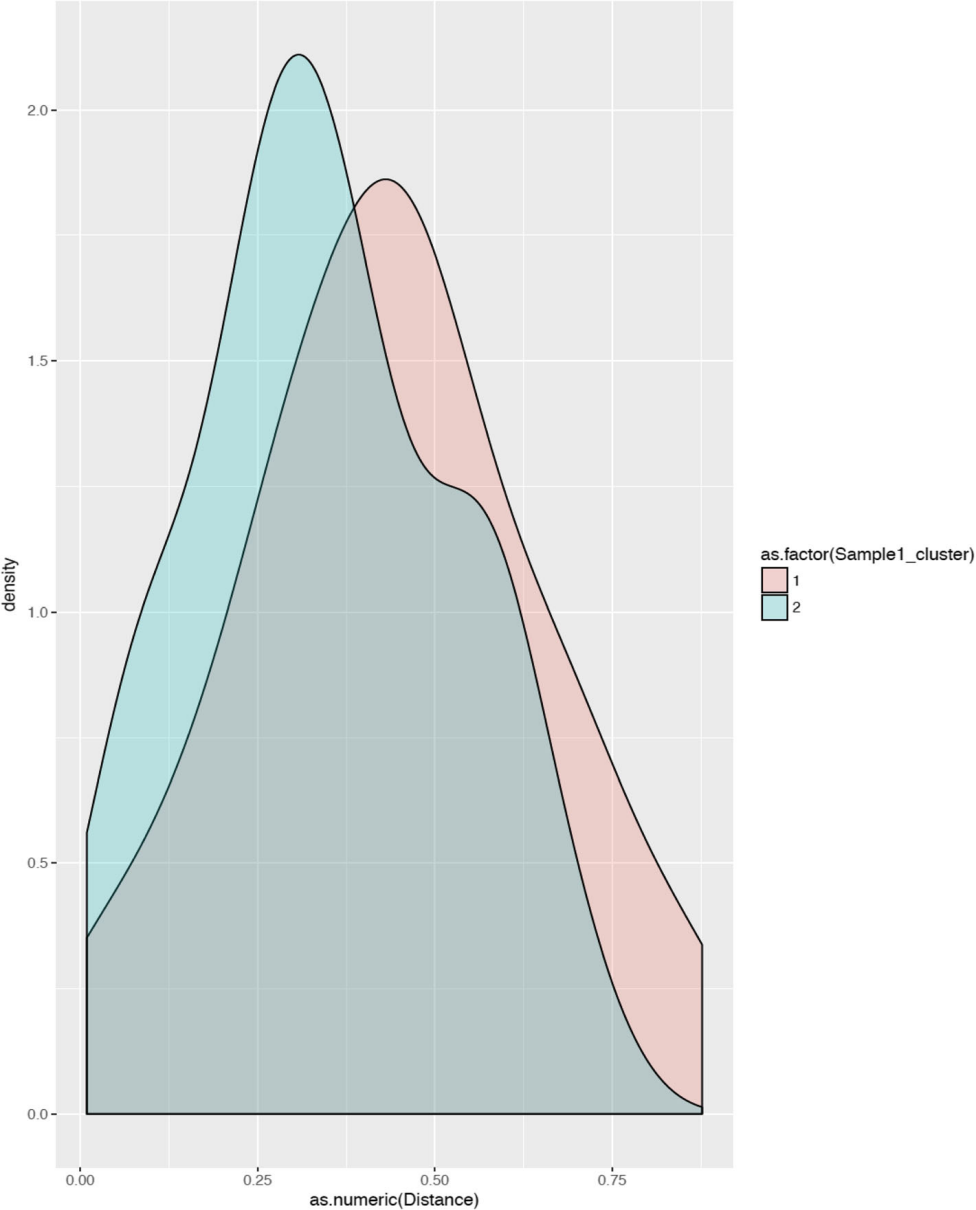
Volatility analysis of the two clusters: Histogram shows the distribution of unweighted UniFrac distances between successive time-points. A distance of 1 means maximally different communities, while a distance of 0 implies identical communities, so a curve shifted toward 0 means lower variability between successive time-points. The two clusters showed a significant difference in variability (*p =* 0.038)

Cluster 1 gained Gammaproteobacteria over time (*p* < 0.001, 3rd and 4th weeks compared to ≤ 2 week samples), whereas cluster 2 showed a transient drop in Gammaproteobacteria in the 3rd week before a 4th week rebound (*p* = 0.042; Fig. 3; in Additional file 2: Table S6 a–c). In cluster 2, *Klebsiella* was the most abundant genus (median 96%, range 0–99%; detected in 19/24 infants), and one particular SNV was dominant (60.8%, 0–99.4%). During the 3rd and the 4th weeks, both clusters showed increasing alpha-diversity and comparable Gammaproteobacteria relative abundance (Additional file 2: Tables S6b, c and S8a, b). Beta-diversity comparisons showed greater between-sample diversity in cluster 2 at ≤ 2 weeks, but these differences narrowed over time (Additional file 2: Table S8c). Volatility analysis confirmed a significant difference in variability in unweighted UniFrac distances between the clusters (*p =* 0.038; Fig. 4) (Additional file 3).

**Fig. 5:**
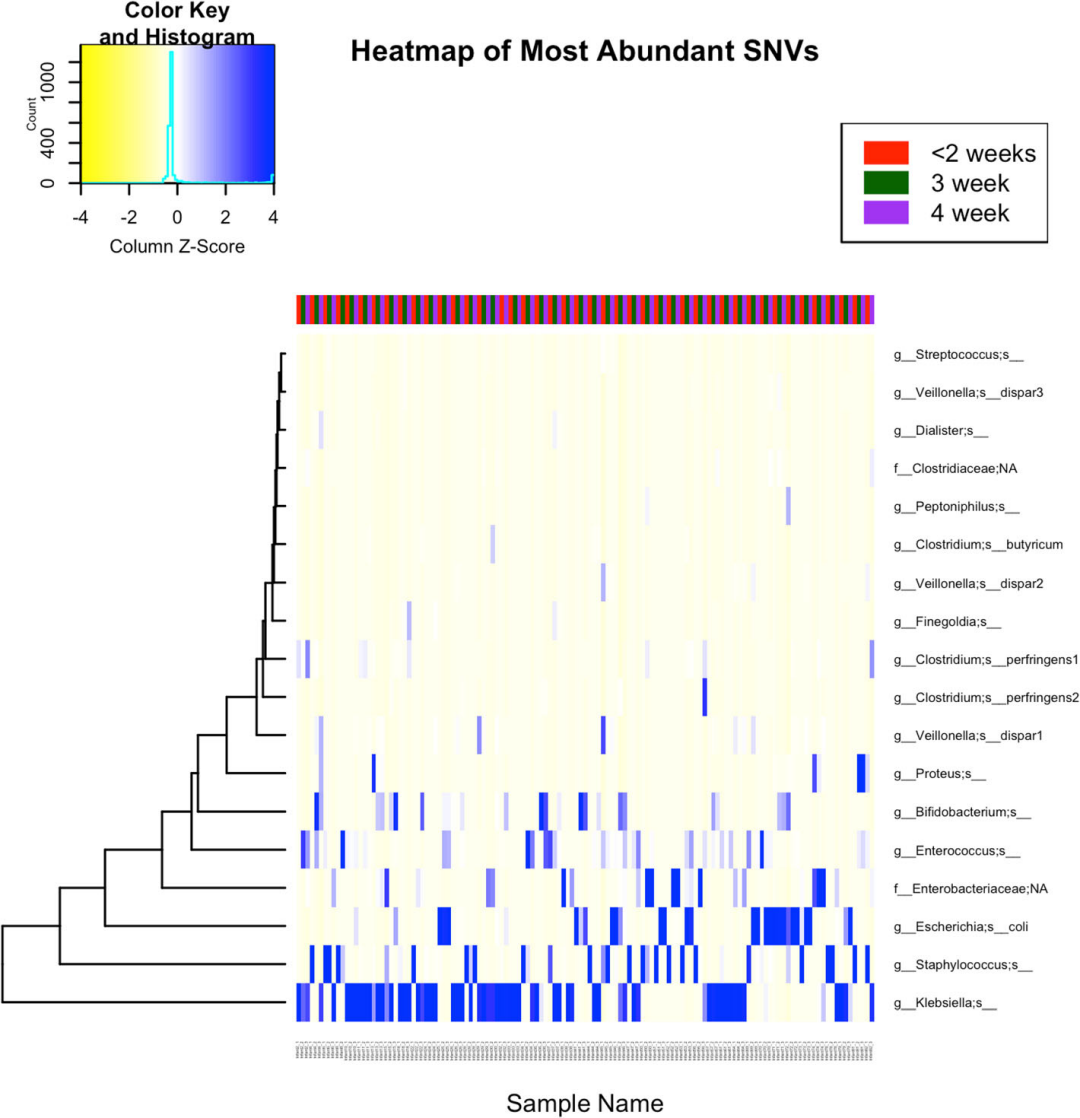
Heat map of the most abundant single nucleotide variants (SNVs): Heat map shows the relative abundance of the 18 most abundant SNVs at each sample. The bar at the top is color coded according to time-point. Blue = most abundant, yellow = least abundant (minimum abundance displayed = 0.165% mean abundance across samples)

ANCOM was performed to identify single nucleotide variant (SNV) sequences that described the variance in the microbiome community differences between clusters 1 and 2. A single variant of *Klebsiella* and a variant of *Staphylococcus* showed significantly different relative abundance between the two clusters (*p* < 0.05; after false discovery rate correction). This same *Staphylococcus* SNV also had a significantly different relative abundance between the three time-points (*p* < 0.001) and decreased over time. No SNVs were significantly different between individual infants. The heat map of the most abundant SNVs in the cohort is shown in Fig. 5.

**Fig. 6:**
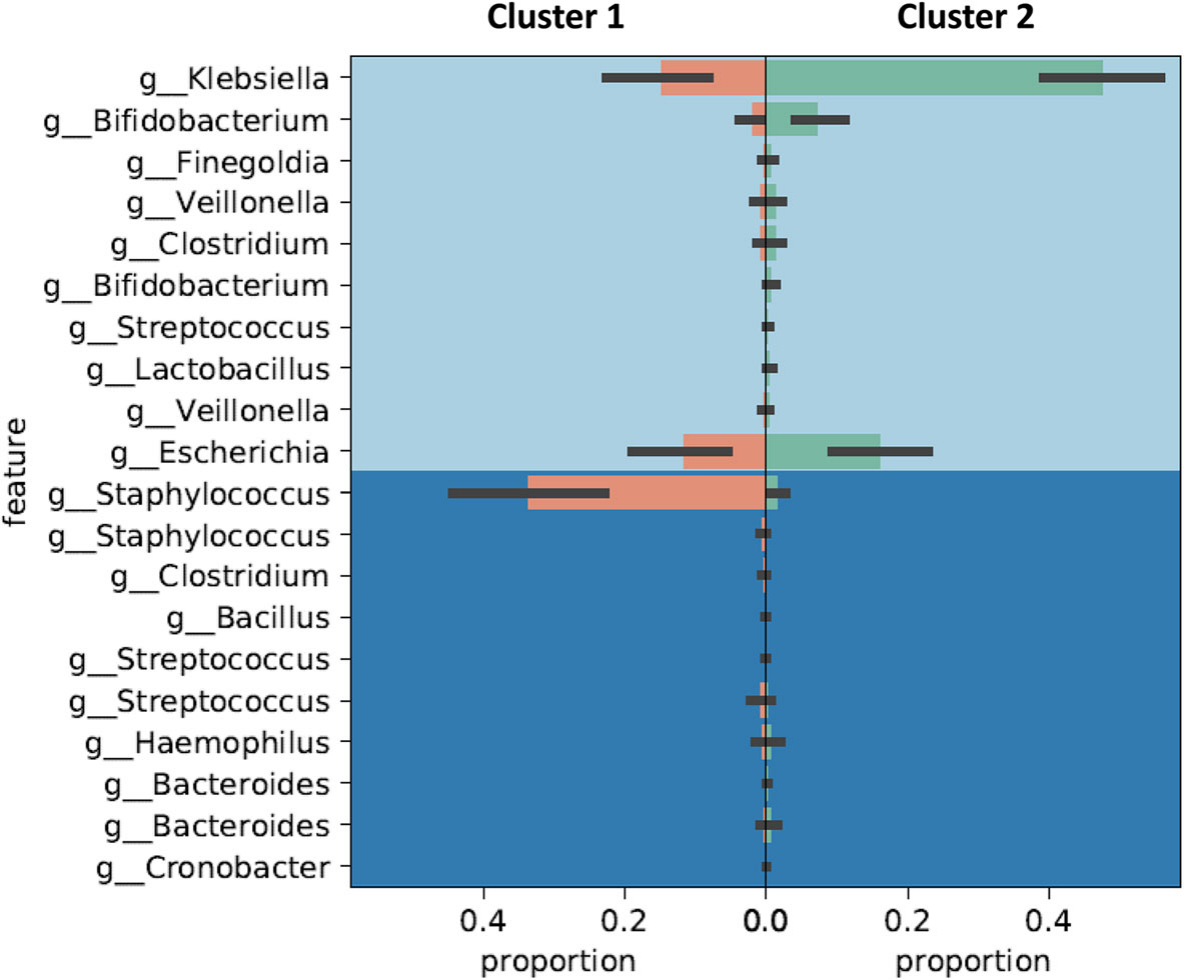
Balance tree analysis for major bacterial taxa. Bifurcating tree relating the DADA2 sequence variants to each other by the time-point for stool collection highlights specific sequence variants that appeared only in early or late stages. Linear regression by cluster membership confirmed increased *Klebsiella* in cluster 2. Cluster 1 showed a true increase in *Staphylococcus* sequence variant

Data on relative abundance/proportions does not inform whether specific taxa have grown or decreased in absolute abundance. We performed Balance Tree analysis, which uses the concept of balances to account for the compositional nature of 16S rRNA data. We calculated a bifurcating tree relating the DADA2 sequence variants to each other by time-point (stool number) to determine if certain sequence variants appeared only in early or late stages. We then performed linear regression by cluster membership, which confirmed that cluster 2 showed increased *Klebsiella* (Fig. 6).

### Clinical antecedents of fecal colonization with Gammaproteobacteria

We performed mixed-effects modeling to identify the clinical determinants of the relative abundance of fecal Gammaproteobacteria. Small-for-gestational age (SGA), ethnicity, vaginal birth, antenatal steroids, magnesium sulfate, chorioamnionitis, gender, multiple births, postnatal age, enteral feedings, RDS, PDA, sepsis, and transfusions were defined as fixed effects. Maternal BMI, birth weight, gestation, postmenstrual age at stool collection, duration of ruptured membranes, antibiotic treatment, and the total length of hospital stay were defined as random effects. The best-fitting, parsimonious model (Table 2) showed positive associations of fecal Gammaproteobacteria with vaginal birth (*F* = 9.55, *p* = 0.002) and antenatal steroids (*F* = 4.23, *p* = 0.042). There was also a borderline, negative effect of maternal magnesium sulfate therapy (*F* = 3.87, *p* = 0.051). There was no effect of gestational or postmenstrual age. When we used *Klebsiella* as the dependent variable, the associations with vaginal birth (*F* = 10.91, *p* = 0.001) and antenatal steroids (*F* = 7.29, *p* = 0.008) remained consistent.

**Table 2:** Linear mixed-effects model for the relative fecal abundance of Gammaproteobacteria

Parameter	Estimate	Std. error	*p* value	95% CI		Bootstrapping 95% CI	
				Lower	Upper	Lower	Upper
Intercept	24.06	22.35	0.284	− 20.19	68.32	− 21.98	345.46
Absence of SGA status	1.90	13.28	0.887	− 24.40	28.19	− 72.52	23.13
Cesarean birth	− 23.63	7.65	*0.002*	− 38.77	− 8.49	− 40.33	− 4.88
Absence of Latino ethnicity	6.86	7.67	0.373	− 8.32	22.04	− 7.73	32.45
Absence of antenatal steroids	− 23.57	11.47	*0.042*	− 46.28	− 0.87	− 46.27	14.82
Absence of antenatal magnesium sulfate therapy	15.38	7.81	0.051	− 0.09	30.85	− 12.83	33.13
Absence of chorioamnionitis	8.41	6.70	0.212	− 4.85	21.67	− 7.79	25.40
Time	2.71	1.70	0.115	− 0.67	6.08	− 7.67	6.17

* Significant p-values (<0.05) are shown in italics

We next performed linear regression to identify the determinants of Gammaproteobacteria relative abundance at the three time-points of stool collection. At ≤ 2 weeks, Gammaproteobacteria abundance was associated with vaginal birth, Latino ethnicity, postnatal age, and the number of antibiotic days (*r*^2^ = 0.69, *F* = 5.66, *p* < 0.001; see Additional file 2: Table S13). When individual antibiotics were included, gentamicin (*b* = 15.03, SE 5.35, *p* = 0.009), but not ampicillin, showed a significant effect. The regression models were less robust during the 3rd week (*r*^2^ = 0.34, *F* = 3.02, *p* = 0.026), but postnatal age and antenatal steroids continued to show significant effects (see Additional file 2: Table S14). The 4th-week regression models were a better fit (*r*^2^ = 0.68, *F* = 4.26, *p* = 0.001) and showed positive effects of postnatal age, antenatal steroids, respiratory distress syndrome (RDS), and red cell transfusions, and negative effects of magnesium sulfate, admission temperature, and total antibiotic days. Individual antibiotics did not show a significant effect. There was a borderline, positive effect of human milk feedings (*p* = 0.055; see Additional file 2: Table S15).

In cluster 1, mixed modeling showed increased Gammaproteobacteria with postnatal age (*p* = 0.002). Cluster 2 showed increased Gammaproteobacteria with vaginal birth (*p* = 0.019) and antenatal steroids (*p =* 0.001), and negative associations with small-for-gestational-age status (SGA) (*p <* 0.001), Latino ethnicity (*p =* 0.009), and chorioamnionitis (*p =* 0.016; Tables 3 and 4). Regression analysis in cluster 1 at ≤ 2 weeks (*r*^2^ = 0.87, *F* = 8.88, *p* = 0.004) showed increased Gammaproteobacteria with human milk feedings. In cluster 2, patent ductus arteriosus (PDA) had a negative effect (Additional file 2: Table S9a, b).

**Table 3:** Linear mixed-effects model for the relative fecal abundance of Gammaproteobacteria in cluster 1

Parameter	Estimate	Std. error	*p* value	95% CI	
				Lower	Upper
Intercept	− 71.41	44.94	0.118	− 161.47	18.65
Absence of SGA status	− 14.95	30.96	0.631	− 76.99	47.08
Cesarean birth	3.24	21.84	0.883	− 40.53	47.00
Absence of Latino ethnicity	34.19	10.82	*0.003*	12.50	55.89
Absence of antenatal steroids	− 20.38	17.39	0.246	− 55.24	14.47
Absence of antenatal magnesium sulfate therapy	4.56	16.41	0.782	− 28.34	37.45
Absence of chorioamnionitis	5.18	8.50	0.545	− 11.85	22.20
Postnatal age	7.25	2.23	*0.002*	2.78	11.72

* Significant p-values (<0.05) are shown in italics

**Table 4:** Linear mixed-effects model for the relative fecal abundance of Gammaproteobacteria in cluster 2

Parameter	Estimate	Std. error	*p* value	95% CI	
				Lower	Upper
Intercept	885.64	111.30	0.000	667.49	1103.78
Absence of SGA status	− 123.76	20.12	*0.000*	− 163.21	− 84.31
Cesarean birth	− 18.48	7.89	*0.019*	− 33.95	− 3.02
Absence of Latino ethnicity	29.33	11.18	*0.009*	7.36	51.29
Absence of antenatal steroids	− 51.94	14.89	*0.001*	− 81.17	− 22.71
Absence of antenatal magnesium sulfate therapy	− 1.12	8.05	0.889	− 17.09	14.84
Absence of chorioamnionitis	22.26	9.26	*0.016*	4.09	40.43
Postnatal age	− 7.76	1.74	*0.000*	− 11.17	− 4.35

* Significant p-values (<0.05) are shown in italics

### Clinical determinants of the relative proportions of other bacterial phyla

Fecal Firmicutes were positively associated with cesarean birth (*F* = 21.49, *p* < 0.001) and negatively with postnatal age (*F* = 5.08, *p* = 0.026). We attempted, but did not find evidence of clear clustering of subjects based on the relative abundance of Firmicutes, Bacilli, or Clostridia. Comparison of clusters based on Gammaproteobacteria abundance (as described in preceding sections) showed interesting differences in the relative abundance of Firmicutes. Cluster 1 carried more Firmicutes and Bacilli than cluster 2 in the earliest (≤ 2 weeks) and 3rd week stool samples (Additional file 2: Table 6a–c and S10a). In cluster 1, Firmicutes were associated positively with Latino ethnicity and negatively with postnatal age (Additional file 2: Table S10b). Bacilli decreased with postnatal age in cluster 2 (Fig. 5 and Additional file 2: Table S11a–c). *Staphylococcus* was dominant in cluster 1 at ≤ 2 weeks (median 100%, range 2–100%; detected in all infants). Most mapped to one particular SNV (median abundance 98% of all Staphylococcus, range 1.8–100%), which was confirmed in Balance Tree analysis (Fig. 6). Clostridia increased with postnatal age (*F* = 8.81, *p* = 0.004), particularly in cluster 2 (Additional file 2: Table S12a–c).

## Discussion

We present a detailed analysis of the clinical determinants of the proportion of Gammaproteobacteria in the stool of preterm infants. Consistent with our hypothesis, the overall proportion of Gammaproteobacteria increased in stool with postnatal age. However, we noted two distinct patterns: one group started with a low relative abundance of Gammaproteobacteria in early stool samples (≤ 2 weeks) that increased with time, whereas a second group of infants started with a high relative abundance of Gammaproteobacteria that dipped transiently during the 3rd week. By the 4th week, the two groups had similar levels of Gammaproteobacteria. To our knowledge, this is the first study to describe this dichotomy in gut microbiome assembly in premature infants.

The development of the preterm gut microbiome is an area of intense scientific scrutiny. Currently, there are two conceptual models: in the first [38], the microbiome is believed to develop in a non-random, patterned progression where host maturation is important and environmental factors have only a minimal, non-enduring influence on the gut microbiome. In the 2nd model, the environment is key: factors such as the hospital microflora, diet, and antibiotics are believed to fundamentally alter gut microbiome assembly [2, 39–42]. Our findings suggest that both models have merit. Cluster 1 showed the sequential dominance of Bacilli, Gammaproteobacteria, and Clostridia in stool samples collected during the first 2, the 3rd, and the 4th weeks, respectively. These findings were consistent with those of La Rosa et al. [38], except that we did not find effects of gestational or postmenstrual age. These infants began life with a low relative abundance of Gammaproteobacteria but showed greater variability between time-points in our volatility analysis. The low alpha-diversity and high beta-diversity in cluster 1 may be interpreted as a gammaproteobacterial bloom during their NICU stay that transiently crowded out other members of a microbial community. These findings differ from those in cluster 2, who showed high relative abundance of fecal Gammaproteobacteria from the earliest stool sample. The identification of vaginal birth as the leading determinant of stool-associated Gammaproteobacteria in this group suggests that vertical, mother-to-infant transmission of Gammaproteobacteria may contribute to intestinal dysbiosis in some preterm infants. This information is important for clinical practice improvement and infection control measures. In preterm infants, early colonization with Gammaproteobacteria has been generally associated with horizontal transmission of these bacteria in the NICU and selection pressures from antibiotics and diet. However, the possibility of vertical transmission in some infants is novel and indicates a need for additional preventive strategies starting before and during birth.

The dominance of a single variant of *Klebsiella* in cluster 2 infants indicates a common, possibly hospital-derived, source. Women with high-risk pregnancies are often exposed repeatedly to the hospital environment while being monitored/treated for pregnancy complications and are at risk of becoming colonized with hospital microflora. In our cohort, 25/43 (58%) mothers had received inpatient care for ≥ 3 hospital days and 22/43 (51%) had ≥ 2 hospital visits before delivery, mostly for actual or imminent preterm labor. We speculate that cluster 2 infants, who were more likely to have had a vaginal birth, may have received a larger inoculum of Gammaproteobacteria/*Klebsiella* than cluster 1 because of the exposure to maternal microflora in the vaginal, fecal, and cutaneous compartments. We are unable to investigate these possibilities further as we did not collect maternal and hospital environmental samples in the present study. During pregnancy, the vaginal microbiome is dominated by Firmicutes [43, 44]. In women with vaginal dysbiosis, pathobionts such as *Prevotella*, *Sneathia*, *Atopobium*, *Mycoplasma*, and *Gardnerella* can be identified [43, 44], but Gammaproteobacteria are infrequent [45]. The putative microbiome of the placenta and the amniotic fluid includes Gammaproteobacteria [46] and could be a plausible source, but this should affect all infants, regardless of delivery mode. Other potential sources may include exposure to maternal enteric flora during vaginal birth and then to the microbiome of human milk, both of which contain Gammaproteobacteria and often *Klebsiella* in particular [47, 48].

The identification of postnatal age as a determinant of Gammaproteobacteria abundance was consistent with the acquisition of these bacteria from care providers and the hospital environment. However, the transient drop in fecal Gammaproteobacteria we observed in cluster 2 infants during the 3rd postnatal week was contrary to our hypothesis that once established in the relatively uninhabited preterm intestine [49], Gammaproteobacteria would either increase or remain stable, but not decrease, over time. These findings need to be confirmed with quantitative measurements of Gammaproteobacteria abundance, but if validated, would have important implications for evaluating dysbiosis as a predictor of adverse outcomes in VLBW infants [11] as the best timing for measuring Gammaproteobacteria abundance will need to be ascertained.

The association of Latino ethnicity with fecal Gammaproteobacteria may be rooted in genetic factors [50], although there may have been a confounding influence of the type of feedings: 8/9 (88.8%) Latino infants received only human milk vs. 17/36 (47.2%) infants of other ethnic groups (*p* = 0.03). The association of fecal Gammaproteobacteria with antenatal steroids, magnesium sulfate, and the admission temperature is also not easily explained. Interestingly, the effect of antenatal steroids was delayed, seen only in the later stool samples (from the 3rd and 4th weeks). Steroids could alter the host-microbial cross-talk by dampening leukocyte activation and cytokine expression [51–54] or via epigenetic changes in the mucosa [55]. Perinatal exposure to magnesium sulfate alters gut motility and some immune responses [56, 57], but the effects on fecal microbiome need further study. Human milk feedings increased fecal Gammaproteobacteria at ≤ 2 weeks, but had a negative effect later. The early effects may be related to milk-borne Gammaproteobacteria [47], possibly selected under the influence of other factors such as antibiotics. Later, negative effects may reflect the benefits of milk prebiotics [58], but the high prevalence of dysbiosis in our cohort indicates that such protection, at least in hospitalized infants, may be modest. Antibiotics, and gentamicin in particular, were associated with increased Gammaproteobacteria during the early neonatal period, but in the 4th week, antibiotic days had a negative effect. Antibiotics do create an environment that promotes the abundance of Gammaproteobacteria [59], but it is unclear why these effects should change with postnatal age.

The strengths of our study are its prospective design, availability of clinical and laboratory data, and repeated measurements of the gut microbiome. The dataset comes from a NICU with a single-patient room floor plan, which is now the favored NICU design and should be representative of most centers in the USA. Emerging data indicate that the floor plan (patient pods vs. single-patient rooms) may be an important determinant of horizontal microbial spread in the NICU [60]. Our study is constrained by its limited sample size and single study site. In addition, the low incidence of NEC in our cohort is a limitation that prevented us from using NEC as a measured outcome. Our findings of high Gammaproteobacteria abundance in some infants within the first 2 weeks also indicate an opportunity to confirm these findings in meconium, which should contain the original, “at birth” microbiome. Finally, the detection of dysbiosis with predominance of a few bacterial communities does not imply pathogenicity, but needs further evaluation at higher levels of resolution.

## Conclusions

We noted a dichotomous pattern of fecal colonization with Gammaproteobacteria in our cohort of preterm infants; some started with low relative abundance of Gammaproteobacteria and acquired those as a function of postnatal age, whereas others carried these bacteria in high abundance since the early postnatal period. The predominance of a single variant of *Klebsiella* indicated a common, possibly hospital-derived, source. Vaginal birth and antenatal steroids were identified as major determinants of stool-associated Gammaproteobacteria, indicating that vertical, mother-to-infant transmission of Gammaproteobacteria may contribute to intestinal dysbiosis in some infants.

## Additional files

Additional file 1: Code for DADA2. (DOCX 101 kb)

Additional file 2: **Table S1.** Temporal changes in alpha-diversity. **Table S2.** Temporal changes in bacterial phyla. **Table S3.** Temporal changes in major bacterial genera in Gammaproteobacteria. **Table S4**. Temporal changes in major bacterial genera in Firmicutes. **Table S5.** Clinical characteristics of the two clusters. **Table S6**a. Relative abundance of major bacterial phyla at < 2 weeks, by cluster. b. Relative abundance of major bacterial phyla during the 3rd week, by cluster. c. Relative abundance of major bacterial phyla during the 4th week, by cluster. **Table S7**. Random-forest analysis of two clusters. **Table S8**a. Temporal changes in alpha-diversity in cluster 1. b. Temporal changes in alpha-diversity in cluster 2. c. Temporal changes in beta-diversity cluster 2 *vs*. cluster 1. **Table S9**a. Linear regression model for fecal abundance of Gammaproteobacteria in cluster 1 at < 2 weeks. b. Linear regression model for fecal abundance of Gammaproteobacteria in cluster 2 at < 2 weeks. **Table S10**a. Linear mixed-effects model for fecal abundance of Firmicutes. b. Linear mixed-effects model for fecal abundance of Firmicutes in cluster 1. c. Linear mixed-effects model for fecal abundance of Firmicutes in cluster 2. **Table S11**a. Linear mixed-effects model for fecal abundance of Bacilli. b. Linear mixed-effects model for fecal abundance of Bacilli in cluster 1. c. Linear mixed-effects model for fecal abundance of Bacilli in cluster 2. **Table S12**a. Linear mixed-effects model for fecal abundance of Clostridia. b. Linear mixed-effects model for fecal abundance of Clostridia in cluster 1. c. Linear mixed-effects model for fecal abundance of Clostridia in cluster 2. **Table S13.** Linear regression model for fecal abundance of Gammaproteobacteria at < 2 weeks. **Table S14.** Linear regression model for fecal abundance of Gammaproteobacteria during the 3rd week. **Table S15.** Linear regression model for fecal abundance of Gammaproteobacteria during the 4th week. (DOCX 86 kb)

Additional file 3: Unweighted UniFrac distance matrix for Fig. 4. (DOC 63 kb)
