# Supplementary material for: Dichotomous development of the gut microbiome in preterm infants
Source: Microbiome. 2018 Sep 12;6:157. doi: 10.1186/s40168-018-0547-8 (PMC6136210; doi:10.1186/s40168-018-0547-8)
Supplement: Supplementary file 1 — Code for DADA2. (DOCX 101 kb) [file 40168_2018_547_MOESM1_ESM.docx]

**1. DADA 2:**

#Load required packages

library("dada2")

library("ggplot2")

library("ShortRead")

library("phyloseq")

library("msa")

library("phangorn")

library("ggvegan")

#Set path

path<-"~/USF_infants/fwd"

fns <-list.files(path)

#Sort F(R1) and R(R2) files

fns <- sort(list.files(path))

fnFs <- fns[grepl("R1", fns)]

fnFs

fnRs <- fns[grepl("R2", fns)]

fnRs

#Read in names of fastq files, string manipulation to put them in order

fastqs <- fns[grepl(".fastq$", fns)]

fastqs <- sort(fastqs) # Sort ensures forward/reverse reads are in same order

fnFs <- fastqs[grepl("_R1", fastqs)] # Just the forward read files

fnRs <- fastqs[grepl("_R2", fastqs)] # Just the reverse read files

# Get sample names, assuming files named as so: SAMPLENAME_XXX.fastq

sample.names <- sapply(strsplit(fnFs, "_"), `[`, 1)

# Specify the full path to the fnFs and fnRs

fnFs <- file.path(path, fnFs)

fnRs <- file.path(path, fnRs)

#Quality profiles of F and R reads--check first few individuals

plotQualityProfile(fnFs[[1]])

plotQualityProfile(fnFs[[2]])

plotQualityProfile(fnFs[[3]])

# if good quality, just trim last 10 nts

plotQualityProfile(fnRs[[1]])

plotQualityProfile(fnRs[[2]])

plotQualityProfile(fnRs[[3]])

# Make directory and filenames for the filtered fastqs

filt_path <- file.path(path, "filtered")

if(!file_test("-d", filt_path)) dir.create(filt_path)

filtFs <- file.path(filt_path, paste0(sample.names, "_F_filt.fastq.gz"))

filtRs <- file.path(filt_path, paste0(sample.names, "_R_filt.fastq.gz"))

#standard filtering parameters: maxN=0 (DADA2 requires no Ns), truncQ=2 and maxEE=2

#Jointly filters F and R reads

for(i in seq_along(fnFs)) {

fastqPairedFilter(c(fnFs[i], fnRs[i]), c(filtFs[i], filtRs[i]),

truncLen=0,

maxN=0, maxEE=c(2,2), truncQ=2,

compress=TRUE, verbose=TRUE)

}

#Dereplicate the filtered fastq files

derepFs <- derepFastq(filtFs, verbose=TRUE)

derepRs <- derepFastq(filtRs, verbose=TRUE)

# Name the derep-class objects by the sample names

names(derepFs) <- sample.names

names(derepRs) <- sample.names

#Error rate learning

#parametric error model (err) and amplicon dataset will have different error rates

#DADA2 learns its error model from the data itself by alternating estimation of the

#error rates and the composition of the sample until they converge

dadaFs.lrn <- dada(derepFs, err=NULL, selfConsist = TRUE, multithread=TRUE)

#Convergence after 5 rounds.

errF <- dadaFs.lrn[[1]]$err_out

dadaRs.lrn <- dada(derepRs, err=NULL, selfConsist = TRUE, multithread=TRUE)

errR <- dadaRs.lrn[[1]]$err_out

#Convergence after 5 rounds.

#visualize the estimated error rates

plotErrors(dadaFs.lrn[[1]], nominalQ=TRUE)

plotErrors(dadaRs.lrn[[1]], nominalQ=TRUE)

#infer sequence variants in each sample

dadaFs <- dada(derepFs, err=errF, multithread=TRUE)

dadaRs <- dada(derepRs, err=errR, multithread=TRUE)

#look at the dada class object

dadaFs[[1]]

#help("dada-class")

# OUTPUT

# dada-class: object describing DADA2 denoising results

#dada-class: object describing DADA2 denoising results

#50 sample sequences were inferred from 7152 input unique sequences.

#Key parameters: OMEGA_A = 1e-40, BAND_SIZE = 16, USE_QUALS = TRUE

#Merge paired reads

#Spurious sequence variants can be further reduced by merging these overlapping reads

#Requires F and R reads to be in matching order

mergers <- mergePairs(dadaFs, derepFs, dadaRs, derepRs, verbose=TRUE)

# Inspect the merger data.frame from the first sample

head(mergers[[1]])

#Make Sequence table

seqtab <- makeSequenceTable(mergers[names(mergers) != "Mock"])

dim(seqtab)

# Inspect distribution of sequence lengths

table(nchar(getSequences(seqtab)))

#The sequence table is a matrix with rows corresponding to (and named by) the

# samples, and columns corresponding to (and named by) the sequence variants

#Sequences that are much longer or shorter than expected may be the result

#of non-specific priming, and may be worth removing

#(eg. seqtab2 <- seqtab[,nchar(colnames(seqtab)) %in% seq(250,256)]

seqtab2 <- seqtab[,nchar(colnames(seqtab)) %in% seq(252,253)]

#Remove chimeras

seqtab.nochim <- removeBimeraDenovo(seqtab2, verbose=TRUE)

dim(seqtab.nochim)

sum(seqtab.nochim)/sum(seqtab)

#following chimera removal, you should still have most reads but a majority of

#sequence variants may be removed (that's OK, but if you lost lots of reads, could

#be caused by primer sequences with ambiguous nucleotides that were not removed prior

#store sequence table

saveRDS(seqtab.nochim,"seqtab.nochim.rds")

#Assign taxonomy against greengenes

taxa <- assignTaxonomy(seqtab.nochim, "~/gg_13_8_train_set_97.fa.gz")

write.table(taxa, "tax_table.txt", sep='\t', row.names=TRUE, quote=FALSE)

unname(head(taxa))

#Handoff to phyloseq

setwd("~/USF_Infants/")

samdf <- read.csv("Metadat7.txt", sep='\t',header=TRUE)

rownames(samdf)<-samdf[,1]

mysam <- subset(samdf,rownames(samdf) %in% rownames(seqtab.nochim) )

ps <- phyloseq(otu_table(seqtab.nochim, taxa_are_rows=FALSE),

sample_data(mysam),

tax_table(taxa))

**2. For machine learning**, QIIME 1.9 command was used:

supervised_learning.py -i otu_table.biom -m map.txt -c NEC -o Random_Forest_output --ntree 1000

(where -c is the category of interest, -o gives output folder with confusion matrix, cv probabilities, feature importance scores. Always used 1000 trees, out of bag error type)

**3. ANCOM:**

Code for ANCOM

## Main function: ANCOM(...)

#Remove zeroes from OTU table

#in QIIME1.9: filter_otus_from_otu_table.py -i seq_table_meta.biom -o seq_tab_nozero.biom -s 10

#output was 20 most enriched OTUs

## Subsidiary function: ancom.detect(...)

############################################################

#### Data Format

# #########################################

# 1.Enter data with first p columns representing OTUs/ Taxa abundance counts

# and last column representing the grouping variable.

# Example:

# OTU1 OTU2 ... OTUp grp

# c11 c12 ... c1p Grp1

# c21 c22 ... c2p Grp2

# ....

#Set working directory

setwd("~/ANCOM/")

#For each variable, make new otu_data default

group<-read.csv("ancom_metadata_bfexclus_1.txt",sep='\t',header = TRUE)

otu_data<-merge(data,group,by="SampleID")

#group<-read.csv("ancom_metadata_delmeth_1.txt",sep='\t',header = TRUE)

#otu_data<-merge(data,group,by="SampleID")

#group<-read.csv("ancom_metadata_maternal_abx_1.txt",sep='\t',header = TRUE)

#otu_data<-merge(data,group,by="SampleID")

#group<-read.csv("ancom_metadata_inftod.txt",sep='\t',header = TRUE)

#otu_data<-merge(data,group,by="SampleID")

#group<-read.csv("ancom_metadata_stoolnumb.txt",sep='\t',header = TRUE)

#otu_data<-merge(data,group,by="SampleID")

### Taxonomy matrix if needed

# 2.Enter taxonomy matrix for the OTUs with each row for a single OTU

# in same order as columns in the real data. Example below:

# OTU_ID Taxonomy

# OTU1 String1

# OTU2 String2

# ...

###########################################

############################################################

############################################################

## The function to detect OTUs/Taxa (Used within the main ANCOM function)

## Arguments:

## otu_data: Data (as described earlier)

## n_otu: Number of OTUs/Taxa (essentially number of columns - 1)

## alpha: significance level

## multcorr: takes value 1 (Stringent correction: As described in the paper)

## OR 2 (Less stringent: Multiplicity corrections are made within OTU/taxa)

## OR 3 (no multiple testing correction used).

otu_data=data

rownames(otu_data)<-otu_data[,1]

n_otu=length(otu_data)-1

#p value

alpha = 0.05

#Set multiple correction (FDR)

multicorr = 1

ancom.detect <- function(otu_data,n_otu,alpha,multcorr,wilcox=FALSE){

logratio.mat=matrix(NA,nr=n_otu,nc=n_otu)

for(i in 1:(n_otu-1)){

for(j in (i+1):n_otu){

data.pair=otu_data[,c(i,j,n_otu+1)]

lr=log((0.001+as.numeric(data.pair[,1]))/(0.001+as.numeric(data.pair[,2])))

logratio.mat[i,j]=wilcox.test(lr[data.pair$grp==unique(data.pair$grp)[1]],

lr[data.pair$grp==unique(data.pair$grp)[2]],

exact=wilcox)$p.value

}

}

ind <- lower.tri(logratio.mat)

logratio.mat[ind] <- t(logratio.mat)[ind]

logratio.mat[which(is.finite(logratio.mat)==FALSE)]=1

mc.pval=t(apply(logratio.mat,1,function(x){

s=p.adjust(x, method = "BH")

return(s)

}))

a=logratio.mat[upper.tri(logratio.mat,diag=F)==T]

b=matrix(0,nc=n_otu,nr=n_otu)

b[upper.tri(b)==T]=p.adjust(a, method = "BH")

diag(b)=NA

ind.1 <- lower.tri(b)

b[ind.1] <- t(b)[ind.1]

if(multcorr==2){

W=apply(mc.pval,1,function(x){

subp=length(which(x<alpha))

})

}else if(multcorr==1){

W=apply(b,1,function(x){

subp=length(which(x<alpha))

})

}else if(multcorr==3){

W=apply(logratio.mat,1,function(x){

subp=length(which(x<alpha))

})

}

return(W)

}

############################################################

############################################################

############################################################

### How to implement ?

############################################################

## The main ANCOM function

## Arguments:

## filepath: Character string of filepath (for data as described earlier)

## Example: "~/Data Repository/data.txt"

## sig: significance level (usually FDR chosen as 0.05)

## multcorr_type: multcorr (defined above).

############################################################

############################################################

ANCOM <- function(real.data,sig,multcorr_type,wilcox){

####real.data <- read.delim(filepath,header=TRUE)

colnames(real.data)[ ncol(real.data) ] <- "grp"

real.data <- data.frame(real.data[which(is.na(real.data$grp)==FALSE),],row.names=NULL)

par1_new=dim(real.data)[2]-1

W.detected <- ancom.detect(real.data,par1_new,sig,multcorr_type,wilcox)

if( ncol(real.data) < 10 ){

### Detected using arbitrary cutoff

results <- colnames(real.data)[which(W.detected > par1_new-1 )]

} else{

### Detected using a stepwise mode detection

if(max(W.detected)/par1_new >= 0.10){

c.start <- max(W.detected)/par1_new

cutoff <- c.start-c(0.05,0.10,0.15,0.20,0.25)

prop_cut<- rep(0,length(cutoff))

for(cut in 1:length(cutoff)){

prop_cut[cut] <- length(which(W.detected>=par1_new*cutoff[cut]))/length(W.detected)

}

del=rep(0,length(cutoff)-1)

for(i in 1:(length(cutoff)-1)){

del[i]=abs(prop_cut[i]-prop_cut[i+1])

}

if(del[1]<0.02&del[2]<0.02&del[3]<0.02){nu=cutoff[1]

}else if(del[1]>=0.02&del[2]<0.02&del[3]<0.02){nu=cutoff[2]

}else if(del[2]>=0.02&del[3]<0.02&del[4]<0.02){nu=cutoff[3]

}else{nu=cutoff[4]}

up_point <- min(W.detected[which(W.detected>=nu*par1_new)])

W.detected[W.detected>=up_point]=99999

W.detected[W.detected<up_point]=0

W.detected[W.detected==99999]=1

results=colnames(real.data)[which(W.detected==1)]

} else{

W.detected=0

results <- "No significant OTUs detected"

}

}

results <- as.data.frame( results , ncol=1 )

colnames(results)= paste0("OTU Significant at FDR = ", sig )

return(results)

}

ANCOM(real.data = otu_data, sig=alpha, multcorr_type = multicorr, wilcox=FALSE)

**4. Gneiss with QIIME2**

#Create gneiss environment within QIIME2

conda create -n gneiss_env gneiss

#Import DADA2 biom file (HDF5), taxonomy, tree, and metadata

$ qiime tools import --input-path dada2/seq_table.biom --type FeatureTable[Frequency] --output-path seq_tab.biom.qza

qiime tools import --input-path tree.tre --output-path tree.qza --type 'Phylogeny[Unrooted]'

$ qiime tools import --input-path dada2/tax_table_cat.txt --output-path taxa.qza --type FeatureData[Taxonomy] --source-format HeaderlessTSVTaxonomyFormat

#Filter out rare OTUs to prevent taking log of zero

$ qiime feature-table filter-features --i-table seq_tab.biom.qza --o-filtered-table seq_tab_filt.qza --p-min-frequency 100

Saved FeatureTable[Frequency] to: seq_tab_filt.qza

#Changes 0s to 1 with pseudocounts

$ qiime composition add-pseudocount --i-table seq_tab_filt.qza --p-pseudocount 1 --o-composition-table composition.qza

Saved FeatureTable[Composition] to: composition.qza

#Generate hierarchy based on stool number (gradient clustering)

$ qiime gneiss gradient-clustering --i-table seq_tab_filt.qza --m-gradient-file tina_map.txt --m-gradient-column stoolNumb --o-clustering stoolnumb_tree.qza --p-weighted

Saved Hierarchy to: stoolnumb_tree.qza

#Make heatmap by stool number

$ qiime gneiss dendrogram-heatmap --i-table composition.qza --i-tree stoolnumb_tree.qza --m-metadata-file ../tina_map_qiime2.txt --m-metadata-column "stoolNumb" --o-visualization "stoolnumb_heatmap" --verbose

Saved Visualization to: stoolnumb_heatmap.qzv

#Create balances

$ qiime gneiss ilr-transform --i-table composition.qza --i-tree stoolnumb_tree.qza --o-balances balances.qza

Saved FeatureTable[Balance] to: balances.qza

#Linear regression

$ qiime gneiss ols-regression --p-formula "MOM_ID+stoolNumb+Cluster" --i-table balances.qza --i-tree stoolnumb_tree.qza --m-metadata-file ../tina_map_qiime2.txt --o-visualization regression_summary.qzv

Saved Visualization to: regression_summary.qzv

#Assign taxonomy to balances

$ qiime gneiss balance-taxonomy --i-table composition.qza --i-tree stoolnumb_tree.qza --i-taxonomy taxa.qza --p-taxa-level 2 --p-balance-name 'y0' --m-metadata-file ../tina_map_qiime2.txt --m-metadata-column stoolNumb --o-visualization y0_taxa_sum.qzv

Saved Visualization to: y0_taxa_sum.qzv

#Summarize taxonomy of balances by Cluster

$ qiime gneiss balance-taxonomy --i-table composition.qza --i-tree stoolnumb_tree.qza --i-taxonomy taxa.qza --p-taxa-level 2 --p-balance-name 'y0' --m-metadata-file ../tina_map_qiime2.txt --m-metadata-column Cluster --o-visualization y0_taxa_sum_cluster.qzv
