# Supplementary material for: Dichotomous development of the gut microbiome in preterm infants
Source: Microbiome. 2018 Sep 12;6:157. doi: 10.1186/s40168-018-0547-8 (PMC6136210; doi:10.1186/s40168-018-0547-8)
Supplement: Supplementary file 2 — Table S1. Temporal changes in alpha-diversity. Table S2. Temporal changes in bacterial phyla. Table S3. Temporal changes in major bacterial genera in Gammaproteobacteria. Table S4. Temporal changes in major bacterial genera in Firmicutes. Table S5. Clinical characteristics of the two clusters. Table S6a. Relative abundance of major bacterial phyla at < 2 weeks, by cluster. b. Relative abundance of major bacterial phyla during the 3rd week, by cluster. c. Relative abundance of major bacterial phyla during the 4th week, by cluster. Table S7. Random-forest analysis of two clusters. Table S8a. Temporal changes in alpha-diversity in cluster 1. b. Temporal changes in alpha-diversity in cluster 2. c. Temporal changes in beta-diversity cluster 2 vs. cluster 1. Table S9a. Linear regression model for fecal abundance of Gammaproteobacteria in cluster 1 at < 2 weeks. b. Linear regression model for fecal abundance of Gammaproteobacteria in cluster 2 at < 2 weeks. Table S10a. Linear mixed-effects model for fecal abundance of Firmicutes. b. Linear mixed-effects model for fecal abundance of Firmicutes in cluster 1. c. Linear mixed-effects model for fecal abundance of Firmicutes in cluster 2. Table S11a. Linear mixed-effects model for fecal abundance of Bacilli. b. Linear mixed-effects model for fecal abundance of Bacilli in cluster 1. c. Linear mixed-effects model for fecal abundance of Bacilli in cluster 2. Table S12a. Linear mixed-effects model for fecal abundance of Clostridia. b. Linear mixed-effects model for fecal abundance of Clostridia in cluster 1. c. Linear mixed-effects model for fecal abundance of Clostridia in cluster 2. Table S13. Linear regression model for fecal abundance of Gammaproteobacteria at < 2 weeks. Table S14. Linear regression model for fecal abundance of Gammaproteobacteria during the 3rd week. Table S15. Linear regression model for fecal abundance of Gammaproteobacteria during the 4th week. (DOCX 86 kb) [file 40168_2018_547_MOESM2_ESM.docx]

**Additional file 2**

**Table S1: Temporal changes in alpha-diversity metrics**

**Table S2. Temporal changes in bacterial communities at the phylum level**

**Table S3. Temporal changes in major bacterial genera in the class Gammaproteobacteria**

**Table S4. Temporal changes in major bacterial genera in Firmicutes**

**Table S5: Clinical Characteristics of the Two Clusters**

**Table S6a. Relative abundance of major bacterial phyla at <2 weeks, by cluster**

**Table S6b. Relative abundance of major bacterial phyla during the 3rd week, by cluster**

**Table S6c. Relative abundance of major bacterial phyla during the 4th week, by cluster**

**Table S7: Random-forest analysis of two clusters**

**Table S8a. Temporal changes in alpha-diversity metrics in cluster 1**

**Table S8b. Temporal changes in alpha-diversity metrics in cluster 2**

**Table S8c. Temporal changes in beta-diversity metrics comparing cluster 2 *vs*. cluster 1**

**Table S9a. Linear regression model for the relative fecal abundance of Gammaproteobacteria in cluster 1 at <2 weeks**

**Table S9b. Linear regression model for the relative fecal abundance of Gammaproteobacteria in cluster 2 at <2 weeks**

**Table S10a. Linear mixed-effects model for the relative fecal abundance of Firmicutes in premature infants**

**Table S10b. Linear mixed-effects model for the relative fecal abundance of Firmicutes in cluster 1**

**Table S10c. Linear mixed-effects model for the relative fecal abundance of Firmicutes in cluster 2**

**Table S11a. Linear mixed-effects model for the relative fecal abundance of Bacilli in premature infants**

**Table S11b. Linear mixed-effects model for the relative fecal abundance of Bacilli in cluster 1**

**Table S11c. Linear mixed-effects model for the relative fecal abundance of Bacilli in cluster 2**

**Table S12a. Linear mixed-effects model for the relative fecal abundance of Clostridia in premature infants**

**Table S12b. Linear mixed-effects model for the relative fecal abundance of Clostridia in cluster 1**

**Table S12c. Linear mixed-effects model for the relative fecal abundance of Clostridia in cluster 2**

**Table S13. Linear regression model for the relative fecal abundance of Gammaproteobacteria in premature neonates in stool samples collected at <2 weeks**

**Table S14. Linear regression model for the relative fecal abundance of Gammaproteobacteria in premature neonates in stool samples collected during the 3rd week**

**Table S15. Linear regression model for the relative fecal abundance of Gammaproteobacteria in premature neonates in stool samples collected during the 4^th^ week**

**Table S1: Temporal changes in alpha-diversity metrics**

| **Alpha diversity metric** | **<2 weeks** | **3rd week** | **4th week** |
| --- | --- | --- | --- |
| Number of OTUs | 22.85 (18.46-31.64) | 30.77 (25.21-40.88)*** | 33.05 (27.26-40.48)*** |
| Shannon index | 0.63 (0.22-1.67) | 1.3 (0.7-1.79)*** | 1.49 (0.05-2.13)*** |
| Chao1 index | 28.33 (22.71-39.86) | 36.4 (32.62-51.54)*** | 42.83 (31.41-49.75) *** |
| Simpson index | 0.18 (0.04-0.52) | 0.34 (0.19-0.55)** | 0.44 (0.3-0.65)*** |
| Phylodiversity | 0.74 (0.48-1.03) | 1.2 (0.8-1.55)*** | 1.14 (1.01-1.41)*** |

Repeated measures analysis of variance. *** p*<0.01, **** p*<0.001 *vs*. the <2-week sample.

**Table S2. Temporal changes in bacterial communities at the phylum level**

| **Bacteria taxonomic unit** | **1^st^ stool sample**  ***median (IQR)*** | **2^nd^ stool sample**  ***median (IQR)*** | **3^rd^ stool sample**  ***median (IQR)*** |
| --- | --- | --- | --- |
| Proteobacteria | 46 (0-90) | 83.5 (54.8-93.3)*** | 77 (57-88.3)*** |
| Gammaproteobacteria | 42.5 (0-90) | 69.7 (29.9-86.9)*** | 75.5 (54.5-86)*** |
| Firmicutes | 41.5 (3.3-100) | 12 (6-25)*** | 14 (6-26.5)*** |
| Bacilli | 100 (60-100) | 51.0 (10.5-96.5)*** | 42 (11-75.5)*** |
| Clostridia | 0 (0-10.3) | 46 (3-86.5) | 42 (8.5-85)*** |
| Actinobacteria | 0 | 0 | 0 (0-13)** |
| Bacterioidetes | 0 | 0 | 0 |

Repeated measures analysis of variance. *** p*<0.01, **** p*<0.001 *vs*. the <2-week samples

**Table S3.Temporal changes in major bacterial genera in the class Gammaproteobacteria**

| **Bacteria genera** | **<2 weeks; median (range)** | **3rd week; median (range)** | **4th week; median (range)** | **p-value** |
| --- | --- | --- | --- | --- |
|  |  |  |  |  |
| *Klebsiella* | 44 (0-100) | 85 (0-99) | 78.5 (0-99) | NS |
| *Haemophilus* | 0 (0-100) | 0 (0-99) | 0 (0-1) | NS |
| *Proteus* | 0 (0-100) | 0 (0-99) | 0 (0-99) | NS |
| *Acinetobacter* | 0 (0-20) | 0 (0-4) | 0 (0-50) | NS |
| *Vibrio* | 0 (0-8) | 0.00 | 0 (0-7) | NS |
| *Pseudomonas* | 0 (0-67) | 0 (0-1) | 0 (0-1) | NS |
| *Enhydrobacter* | 0 (0-7) | 0 (0-5) | 0 (0-1) | NS |
| *Salmonella* | 0 (0-3) | 0 | 0 (0-2) | NS |
| *Microbulbifer* | 0 (0-8) | 0 | 0 | NS |
| *Trabulsiella* | 0 (0-1) | 0 (0-3) | 0 (0-2) | NS |
| *Arenimonas* | 0 (0-6) | 0.00 | 0 | NS |
| *Photobacterium* | 0 | 0 (0-4) | 0 | NS |
| *Cardiobacterium* | 0 | 0 (0-1) | 0 | NS |
| *Serratia* | 0 | 0 (0-2) | 0 | NS |
| *Stenotrophomonas* | 0 | 0 (0-1) | 0 | NS |
| *Morganella* | 0 | 0 | 0 (0-64) | NS |
| *Erwinia* | 0 (0-34) | 0 (0-99) | 0 (0-26) | NS |

Repeated measures analysis of variance.

**Table S4. Temporal changes in major bacterial genera in Firmicutes**

| **Bacteria Genera** | **<2 weeks; median (range)** | **3rd week; median (range)** | **4th week; median (range)** |  |
| --- | --- | --- | --- | --- |
|  |  |  |  | **p-value** |
| *Veillonella* | 0 (0-83) | 0 (0-97) | 1 (0-88) | **0.004** |
| *Staphylococcus** | 59.5 (0-100) | 2 (0-100) | 0 (0-100) | **<0.001** |
| *Streptococcus* | 0 (0-61) | 0 (0-50) | 0 (0-83) | NS |
| *Enterococcus* | 0 (0-99) | 11 (0-92) | 19 (0-99) | **0.007** |
| *Granulicatella* | 0 (0-7) | 0 (0-26) | 0 (0-3) | **0.051** |
| *Clostridium* | 0 (0-95) | 0 (0-21) | 0 (0-74) | NS |
| *Lactobacillus* | 0 (0-49) | 0 (0-99) | 0 (0-41) | NS |
| *Anaerococcus* | 0 (0-2) | 0 (0-76) | 0 (0-27) | NS |
| *Finegoldia* | 0 (0-3) | 0 (0-29) | 0 (0-47) | NS |
| *Peptoniphilus* | 0 (0-43) | 0 (0-20) | 0 (0-22) | NS |
| *Dialister* | 0 (0-45) | 0 (0-25) | 0 (0-32) | NS |
| *Lactococcus* | 0 | 0 (0-37) | 1. (0-5) | NS |

*A single sequence variant comprised 7.5%, range 0-100% at <2 weeks, 0.2% (0-90.2%) in the 3^rd^ (*p* <0.001), and 0.1 (0-99.9%) of all reads in the 4^th^ week (*p* <0.001). Repeated measures analysis of variance.

**Table S5: Clinical characteristics of the two clusters**

| **Characteristic** | **Cluster 1 (n=20)** | **Cluster 2 (n=24)** | **p value** |
| --- | --- | --- | --- |
| Gestational age, mean (SD), weeks | 28.0 (2.6) | 27.9 (1.9) | 0.855 |
| Birth weight, mean (SD), grams | 1053 (226) | 1176 (175) | **0.049** |
| Male (%) | 9 (45) | 11 (46) | 1.000 |
| Hispanic ethnicity (%) | 3 (15) | 6 (25) | 0.477 |
| Race  Black  White | 9 (45)  11 (55) | 10 (42)  13 (54) | 0.650  0.650 |
| Antenatal medications (%)  Steroids  Magnesium sulfate | 17 (85)  17 (85) | 22 (92)  17 (71) | 0.646  0.306 |
| Vaginal birth (%) | 1 (5 ) | 10 (42) | **0.006** |
| Multiple births (%) | 5 (25) | 2 (8) | 0.217 |
| Chorioamnionitis (%) | 12 (60) | 13 (54) | 0.757 |
| Maternal hypertension (%) | 7 (35) | 6 (25) | 0.522 |
| Small for gestational age (%) | 1 (5) | 2 (8) | 1.000 |
| Respiratory distress syndrome (%) | 12 (60) | 13 (54) | 0.766 |
| Oxygen at 28 days (%) | 5 (25) | 5 (21) | 0.511 |
| Oxygen at 36 weeks (%) | 1 (5) | 1 (4) | 1.000 |
| Patent ductus arteriosus (%) | 3 (15) | 2 (8) | 0.646 |
| Indomethacin (%) | 1 (5) | 1 (4) | 1.000 |
| Patent ductus arteriosus ligation (%) | 1 (5) | 0 | 0.455 |
| Retinopathy of prematurity required treatment (%) | 0 | 0 | 1.000 |
| Necrotizing enterocolitis (%) | 1 (5) | 0 | 0.455 |
| Surgical necrotizing enterocolitis (%) | 1 (5) | 0 | 0.455 |
| Days on antibiotic, mean (SD) | 5 (5) | 4 (5) | 0.753 |
| Positive blood culture (%) | 3 (15) | 2 (8) | 0.646 |
| Packed red blood cell transfusion (%) | 6 (30) | 9 (38) | 0.752 |
| Feeding type (%)  Maternal breast milk only  Formula only  Mixed feeding types | 11 (55)  2 (10)  7 (35) | 13 (54)  0  11 (46) | 0.257  0.257  0.257 |
| Discharge weight <10^th^ percentile (%) | 7 (75) | 4 (17) | 0.186 |
| Length of stay, mean (SD), days | 74 (35) | 63 (22) | 0.218 |

Student’s t test, Fisher’s exact test.

**Table S6a. Relative abundance of major bacterial phyla at ≤2 weeks, by cluster**

| **Bacterial community and subject cluster** | | **Mean** | **Std. Deviation** | **Std. Error Mean** | **p-value** |
| --- | --- | --- | --- | --- | --- |
| Gammaproteobacteria | 1 | 2.0930 | 5.91984 | 1.32372 | **<0.001** |
|  | 2 | 79.1817 | 21.59788 | 4.40865 |  |
| Firmicutes | 1 | 96.95 | 6.825 | 1.526 | **<0.001** |
|  | 2 | 13.29 | 15.622 | 3.189 |  |
| Bacilli | 1 | 92.5500 | 23.85367 | 5.33384 | **0.019** |
|  | 2 | 68.1508 | 40.95730 | 8.36037 |  |
| Clostridia | 1 | 7.4000 | 23.65854 | 5.29021 | NS |
|  | 2 | 20.3496 | 32.01494 | 6.53502 |  |
| Actinobacteria | 1 | 0.05 | 0.224 | 0.050 | NS |
|  | 2 | 3.88 | 10.489 | 2.141 |  |

Mann-Whitney *U* test

**Table S6b. Relative abundance of major bacterial phyla during the 3rd week, by cluster**

| **Bacterial community and subject cluster** | | **Mean** | **Std. Deviation** | **Std. Error Mean** | **p-value** |
| --- | --- | --- | --- | --- | --- |
| Gammaproteobacteria | 1 | 48.4780 | 41.70301 | 9.56733 | NS |
|  | 2 | 65.0586 | 26.50230 | 5.65031 |  |
| Firmicutes | 1 | 37.63 | 39.181 | 8.989 | **0.015** |
|  | 2 | 12.87 | 12.487 | 2.604 |  |
| Bacilli | 1 | 74.20 | 32.94589 | 7.36692 | **0.005** |
|  | 2 | 42.4583 | 37.87046 | 7.73028 |  |
| Clostridia | 1 | 25.80 | 32.94589 | 7.36692 | **0.017** |
|  | 2 | 53.3750 | 38.49202 | 7.85715 |  |
| Actinobacteria | 1 | 3.11 | 12.600 | 2.891 | NS |
|  | 2 | 4.61 | 8.441 | 1.760 |  |

Mann-Whitney *U* test

**Table S6c. Relative abundance of major bacterial phyla during the 4th week, by cluster**

| **Bacterial community and patient cluster** | | **Mean** | **Std. Deviation** | **Std. Error Mean** | **p-value** |
| --- | --- | --- | --- | --- | --- |
| Gammaproteobacteria | 1 | 59.9500 | 35.77337 | 7.99917 | NS |
|  | 2 | 73.6957 | 18.53359 | 3.86452 |  |
| Firmicutes | 1 | 31.30 | 34.345 | 7.680 | NS |
|  | 2 | 15.54 | 13.201 | 2.695 |  |
| Bacilli | 1 | 50.7000 | 39.70563 | 8.87845 | NS |
|  | 2 | 43.4583 | 32.04071 | 6.54028 |  |
| Clostridia | 1 | 39.3500 | 38.16209 | 8.53330 | NS |
|  | 2 | 52.2917 | 32.68091 | 6.67096 |  |
| Actinobacteria | 1 | 3.70 | 8.260 | 1.847 | **0.046** |
|  | 2 | 12.54 | 18.875 | 3.853 |  |

Mann-Whitney *U* test

**Table S7: Random-forest analysis of two clusters**

| Model | Random Forest |
| --- | --- |
| Error type | out-of-bag |
| Estimated error | 0.20769 |
| Baseline error (for random guessing) | 0.46212 |
| Ratio baseline error to observed error | 2.22503 |
| Number of trees | 1000 |

**Table S8a. Temporal changes in alpha-diversity metrics in cluster 1**

| **Alpha diversity metric** | **<2 weeks** | **3rd week** | **4th week** |
| --- | --- | --- | --- |
| Number of OTUs | 19.55 (16.04-23.21) | 29.69 (22.74-39.68)* | 31.14 (24.3-35.22) |
| Shannon index | 0.24 (0.14-0.77) | 1.28 (0.62-1.87) | 1.36 (0.82-1.89) |
| Chao index | 24.33 (20.93-29.06) | 36.17 (23.26-56.34) | 40.61 (29.98-44.17) |
| Simpson index | 0.05 (0.03-0.28) | 0.31 (0.14-0.55) | 0.44 (0.28-0.63)* |
| Phylodiversity | 0.7 (0.44-1) | 1.16 (0.64-1.46)* | 1.12 (0.76-1.39)* |

Repeated measures analysis of variance. * *p*<0.05, *** p*<0.01, **** p*<0.001

**Table S8b. Temporal changes in alpha-diversity metrics in cluster 2**

| **Alpha-diversity metric** | **<2 weeks** | **3rd week** | **4th week** |
| --- | --- | --- | --- |
| Number of OTUs | 25.99 (20.35-33.39) | 32.89 (26.1-41.53) | 34.92 (29.84-41.31)* |
| Shannon index | 1.17 (0.53-1.79) | 1.31 (0.73-1.77) | 1.51 (1.19-2.35) |
| Chao index | 31.69 (26.4-41.2) | 36.77 (32.87-51.34) | 43.33 (34.84-40.38)* |
| Simpson index | 0.33 (0.12-0.57) | 0.34 (0.21-0.57) | 0.45 (0.32-0.66) |
| Phylodiversity | 0.78 (0.48-1.16) | 1.35 (0.86-1.6)** | 1.2 (1.06-1.57)** |

Repeated measures analysis of variance **p*<0.05, *** p*<0.01, **** p*<0.001

**Table S8c. Temporal changes in beta-diversity metrics comparing cluster 2 *vs*. cluster 1**

| **Beta-diversity metric** | **Pseudo-f statistic (cluster 2: cluster 1)** | | |
| --- | --- | --- | --- |
|  | **<2 weeks** | **3^rd^ week** | **4^th^ week** |
| Bray-Curtis dissimilarity | 22.136*** | 3.013* | 1.133 |
| Jaccard coefficient | 15.871*** | 2.211* | 1.071 |
| Unweighted UniFrac measure | 2.524 | 1.791 | 0.989 |
| Weighted UniFrac measure | 44.909*** | 2.582* | 0.848 |

PERMANOVA; ** p*<0.05, **** p*<0.001

**Table S9a. Linear regression model for the relative fecal abundance of Gammaproteobacteria in cluster 1 at <2 weeks**

| **Model** | **Unstandardized Coefficients** | | **Standardized Coefficients** | **t** | **Sig.** |
| --- | --- | --- | --- | --- | --- |
|  | **B** | **Std. Error** | **Beta** |  |  |
| (Constant) | 22.834 | 8.875 |  | 2.573 | **0.033** |
| Antenatal steroids | -16.021 | 3.691 | -0.832 | -4.340 | **0.002** |
| Maternal body mass index | -0.327 | 0.139 | -0.413 | -2.351 | **0.047** |
| Enteral feedings (mother's own milk) | 4.501 | 1.686 | 0.343 | 2.670 | **0.028** |
| Surfactant administration | -0.056 | 0.038 | -0.199 | -1.481 | 0.177 |
| Gender | -4.289 | 1.975 | -0.327 | -2.171 | 0.062 |
| Postnatal age | 0.752 | 0.391 | 0.302 | 1.921 | 0.091 |

**Table S9b. Linear regression model for the relative fecal abundance of Gammaproteobacteria in cluster 2 at <2 weeks**

| **Model** | **Unstandardized Coefficients** | | **Standardized Coefficients** | **t** | **Sig.** |
| --- | --- | --- | --- | --- | --- |
|  | **B** | **Std. Error** | **Beta** |  |  |
| (Constant) | 84.200 | 3.837 |  | 21.946 | 0.000 |
| PDA | -43.020 | 12.725 | -0.603 | -3.381 | **0.003** |

**Table S10a. Linear mixed-effects model for the relative fecal abundance of Firmicutes**

| **Parameter** | **Estimate** | **Std. Error** | **Sig.** | **95% Confidence Interval** | |
| --- | --- | --- | --- | --- | --- |
|  |  |  |  | **Lower Bound** | **Upper Bound** |
| Intercept | 65.659 | 21.235 | 0.002 | 23.624 | 107.693 |
| No SGA | 0.022 | 12.693 | 0.999 | -25.104 | 25.148 |
| Cesarean birth | 33.099 | 7.14 | **0.000** | 18.966 | 47.231 |
| Non-Latino ethnicity | -5.334 | 7.322 | 0.468 | -19.828 | 9.159 |
| No antenatal steroids | 16.837 | 10.96 | 0.127 | -4.857 | 38.532 |
| No antenatal magnesium sulfate therapy | -12.202 | 7.422 | 0.103 | -26.892 | 2.489 |
| No chorioamnionitis | -10.839 | 6.401 | 0.093 | -23.509 | 1.831 |
| Postnatal age at stool collection | -3.648 | 1.619 | **0.026** | -6.852 | -0.444 |

**Table S10b. Linear mixed-effects model for the relative fecal abundance of Firmicutes in cluster 1**

| **Parameter** | **Estimate** | **Std. Error** | **Sig.** | **95% CI** | |
| --- | --- | --- | --- | --- | --- |
|  |  |  |  | **Lower** | **Upper** |
| Intercept | 155.53 | 42.57 | 0.001 | 70.21 | 240.85 |
| No SGA | 26.86 | 29.33 | 0.364 | -31.91 | 85.64 |
| Cesarean birth | 6.25 | 20.69 | 0.764 | -35.21 | 47.71 |
| Non-Latino ethnicity | -32.60 | 10.26 | **0.002** | -53.16 | -12.05 |
| No antenatal steroids | 28.60 | 16.48 | 0.088 | -4.42 | 61.62 |
| No antenatal magnesium sulfate therapy | -0.19 | 15.55 | 0.991 | -31.35 | 30.98 |
| No chorioamnionitis | -6.18 | 8.05 | 0.446 | -22.31 | 9.95 |
| Postnatal age at stool collection | -8.36 | 2.11 | **0.000** | -12.60 | -4.13 |

**Table S10c. Linear mixed-effects model for the relative fecal abundance of Firmicutes in cluster 2**

| **Parameter** | **Estimate** | **Std. Error** | **p-value** | **95% CI** | |
| --- | --- | --- | --- | --- | --- |
|  |  |  |  | **Lower** | **Upper** |
| Intercept | -9.22 | 14.62 | 0.596 | -75.24 | 56.80 |
| No SGA | 0.61 | 5.73 | 0.916 | -10.84 | 12.05 |
| Cesarean birth | 6.03 | 4.33 | 0.169 | -2.62 | 14.67 |
| Non-Latino ethnicity | -4.57 | 5.29 | 0.391 | -15.13 | 5.99 |
| No antenatal steroids | -1.17 | 7.90 | 0.883 | -16.94 | 14.60 |
| No antenatal magnesium sulfate therapy | -10.39 | 4.23 | **0.017** | -18.82 | -1.95 |
| No chorioamnionitis | 2.80 | 5.09 | 0.585 | -7.37 | 12.96 |
| Postnatal age at stool collection | 0.97 | 0.88 | 0.275 | -0.79 | 2.72 |

**Table S11a. Linear mixed-effects model for the relative fecal abundance of Bacilli in premature infants**

| **Parameter** | **Estimate** | **Std. Error** | **Sig.** | **95% Confidence Interval** | |
| --- | --- | --- | --- | --- | --- |
|  |  |  |  | **Lower Bound** | **Upper Bound** |
| Intercept | 85.99 | 24.66 | 0.001 | 37.19 | 134.79 |
| No SGA | 20.04 | 14.05 | 0.156 | -7.76 | 47.84 |
| Cesarean birth | 9.08 | 8.36 | 0.280 | -7.47 | 25.63 |
| Non-Latino ethnicity | -6.58 | 8.77 | 0.455 | -23.93 | 10.77 |
| No antenatal steroids | -7.28 | 13.11 | 0.580 | -33.24 | 18.67 |
| No antenatal magnesium sulfate | -5.55 | 8.76 | 0.527 | -22.89 | 11.78 |
| No chorioamnionitis | -1.93 | 7.63 | 0.801 | -17.03 | 13.17 |
| Postnatal age at stool collection | -3.46 | 1.91 | 0.073 | -7.24 | 0.33 |

**Table S11b. Linear mixed-effects model for the relative fecal abundance of Bacilli in cluster 1**

| **Parameter** | **Estimate** | **Std. Error** | **p-value** | **95% CI** | |
| --- | --- | --- | --- | --- | --- |
|  |  |  |  | **Lower** | **Upper** |
| Intercept | -10.15 | 44.68 | 0.821 | -99.65 | 79.35 |
| No SGA | 98.55 | 30.75 | **0.002** | 36.96 | 160.15 |
| Cesarean birth | 25.50 | 21.71 | 0.245 | -17.99 | 69.00 |
| Non-Latino ethnicity | -6.16 | 10.73 | 0.568 | -27.66 | 15.34 |
| No antenatal steroids | -9.56 | 17.28 | 0.582 | -44.19 | 25.06 |
| No antenatal magnesium sulfate | 10.83 | 16.27 | 0.509 | -21.76 | 43.42 |
| No chorioamnionitis | -4.48 | 8.40 | 0.595 | -21.30 | 12.33 |
| Postnatal age at stool collection | -0.40 | 2.20 | 0.856 | -4.80 | 4.00 |

**Table S11c. Linear mixed-effects model for the relative fecal abundance of Bacilli in cluster 2**

| **Parameter** | **Estimate** | **Std. Error** | **p-value** | **95% CI** | |
| --- | --- | --- | --- | --- | --- |
|  |  |  |  | **Lower** | **Upper** |
| Intercept | 117.13 | 32.44 | 0.001 | 52.42 | 181.85 |
| No SGA | -8.39 | 16.11 | 0.604 | -40.53 | 23.74 |
| Cesarean birth | -4.32 | 12.89 | 0.739 | -30.02 | 21.39 |
| Non-Latino ethnicity | 0.94 | 15.57 | 0.952 | -30.12 | 32.00 |
| No antenatal steroids | 14.38 | 24.31 | 0.556 | -34.11 | 62.87 |
| No antenatal magnesium sulfate | -4.64 | 12.00 | 0.700 | -28.58 | 19.31 |
| No chorioamnionitis | 3.90 | 14.97 | 0.795 | -25.97 | 33.76 |
| Postnatal age at stool collection | -6.04 | 2.66 | **0.026** | -11.34 | -0.74 |

**Table S12a. Linear mixed-effects model for the relative fecal abundance of Clostridia in premature infants**

| **Parameter** | **Estimate** | **Std. Error** | **Sig.** | **95% Confidence Interval** | |
| --- | --- | --- | --- | --- | --- |
|  |  |  |  | **Lower Bound** | **Upper Bound** |
| Intercept | -26.54 | 24.17 | 0.279 | -75.59 | 22.50 |
| No SGA | -21.45 | 12.81 | 0.098 | -46.91 | 4.02 |
| Cesarean birth | 3.67 | 7.73 | 0.636 | -11.70 | 19.04 |
| Non-Latino ethnicity | 8.38 | 8.00 | 0.297 | -7.48 | 24.25 |
| No antenatal steroids | 2.12 | 11.60 | 0.855 | -20.84 | 25.09 |
| No antenatal magnesium sulfate | 14.35 | 8.09 | 0.080 | -1.78 | 30.47 |
| No chorioamnionitis | 2.82 | 6.98 | 0.687 | -11.04 | 16.69 |
| Postnatal age at stool collection | 5.01 | 1.69 | **0.004** | 1.67 | 8.35 |

**Table S12b. Linear mixed-effects model for the relative fecal abundance of Clostridia in cluster 1**

| **Parameter** | **Estimate** | **Std. Error** | **p-value** | **95% CI** | |
| --- | --- | --- | --- | --- | --- |
|  |  |  |  | **Lower** | **Upper** |
| Intercept | 98.32 | 42.45 | 0.024 | 13.28 | 183.36 |
| No SGA | -95.30 | 29.22 | **0.002** | -153.83 | -36.78 |
| Cesarean birth | -24.01 | 20.63 | 0.249 | -65.34 | 17.32 |
| Non-Latino ethnicity | 5.27 | 10.20 | 0.608 | -15.16 | 25.70 |
| No antenatal steroids | 7.80 | 16.42 | 0.637 | -25.10 | 40.70 |
| No antenatal magnesium sulfate | -3.49 | 15.46 | 0.822 | -34.45 | 27.48 |
| No chorioamnionitis | 11.21 | 7.98 | 0.165 | -4.77 | 27.19 |
| Postnatal age at stool collection | 1.33 | 2.09 | 0.526 | -2.85 | 5.51 |

**Table S12c. Linear mixed-effects model for the relative fecal abundance of Clostridia in cluster 2**

| **Parameter** | **Estimate** | **Std. Error** | **p-value** | **95% CI** | |
| --- | --- | --- | --- | --- | --- |
|  |  |  |  | **Lower** | **Upper** |
| Intercept | -44.67 | 26.82 | 0.100 | -98.17 | 8.84 |
| No SGA | 4.74 | 13.32 | 0.723 | -21.83 | 31.31 |
| Cesarean birth | 7.60 | 10.65 | 0.478 | -13.65 | 28.85 |
| Non-Latino ethnicity | -12.65 | 12.87 | 0.329 | -38.33 | 13.02 |
| No antenatal steroids | -39.94 | 20.10 | 0.051 | -80.03 | 0.15 |
| No antenatal magnesium sulfate | 9.05 | 9.92 | 0.365 | -10.75 | 28.84 |
| No chorioamnionitis | 3.74 | 12.38 | 0.763 | -20.95 | 28.43 |
| Postnatal age at stool collection | 8.27 | 2.20 | **0.000** | 3.88 | 12.65 |

**Table S13. Linear regression model for the relative fecal abundance of Gammaproteobacteria in stool samples collected at <2 weeks**

| **Model** | **Unstandardized Coefficients** | | **Standardized Coefficients** | **t** | **Sig.** |
| --- | --- | --- | --- | --- | --- |
|  | **B** | **Std. Error** | **Beta** |  |  |
| (Constant) | -355.875 | 302.710 |  | -1.176 | 0.250 |
| Vaginal delivery | 45.135 | 11.850 | 0.497 | 3.809 | **0.001** |
| Chorioamnionitis | -23.077 | 11.486 | -0.273 | -2.009 | 0.055 |
| Admission temperature | 6.869 | 8.542 | 0.101 | 0.804 | 0.429 |
| Postnatal age | 5.160 | 1.862 | 0.377 | 2.772 | **0.010** |
| PDA | -32.157 | 18.892 | -0.241 | -1.702 | 0.101 |
| Early-onset sepsis | -122.451 | 42.438 | -0.479 | -2.885 | **0.008** |
| Latino ethnicity | 41.541 | 17.574 | 0.311 | 2.364 | **0.026** |
| Antibiotics in 1st 2 weeks | 5.884 | 2.824 | 0.345 | 2.084 | **0.047** |
| Antenatal steroids | 34.714 | 18.633 | 0.260 | 1.863 | 0.074 |
| Birth weight | 0.050 | 0.028 | 0.236 | 1.800 | 0.083 |

**Table S14. Linear regression model for the relative fecal abundance of Gammaproteobacteria in stool samples collected during the 3rd week**

| **Model** | **Unstandardized Coefficients** | | **Standardized Coefficients** | **t** | **Sig.** |
| --- | --- | --- | --- | --- | --- |
|  | **B** | **Std. Error** | **Beta** |  |  |
| (Constant) | -31.507 | 31.427 |  | -1.003 | 0.324 |
| Indomethacin | 61.631 | 32.438 | 0.333 | 1.900 | 0.067 |
| Antenatal steroids | 51.272 | 17.904 | 0.582 | 2.864 | **0.008** |
| Antenatal magnesium sulfate | -24.205 | 11.703 | -0.365 | -2.068 | **0.048** |
| Postnatal age at stool collection | 2.885 | 1.343 | 0.364 | 2.148 | **0.040** |
| PDA | 27.481 | 17.077 | 0.312 | 1.609 | 0.118 |

**Table S15. Linear regression model for the relative fecal abundance of Gammaproteobacteria in stool samples collected during the 4^th^ week**

| **Model** | **Unstandardized Coefficients** | | **Standardized Coefficients** | **t** | **Sig.** |
| --- | --- | --- | --- | --- | --- |
|  | **B** | **Std. Error** | **Beta** |  |  |
| (Constant) | 629.840 | 236.836 |  | 2.659 | 0.013 |
| Antenatal steroids | 24.275 | 11.381 | 0.316 | 2.133 | **0.043** |
| Length of hospitalization | -0.577 | 0.150 | -0.640 | -3.858 | **0.001** |
| Postnatal age | 2.207 | 0.828 | 0.389 | 2.665 | **0.013** |
| Admission temperature | -14.986 | 6.104 | -0.327 | -2.455 | **0.021** |
| Maternal body mass index | -1.157 | 0.504 | -0.326 | -2.295 | **0.030** |
| RBC transfusions | 16.611 | 9.012 | 0.311 | 1.843 | 0.077 |
| Total number of antibiotic days | -1.605 | 0.763 | -0.300 | -2.104 | **0.046** |
| Antenatal magnesium sulfate | -23.961 | 8.697 | -0.417 | -2.755 | **0.011** |
| RDS | 19.743 | 8.175 | 0.369 | 2.415 | **0.023** |
| Gender | -14.842 | 7.742 | -0.283 | -1.917 | 0.067 |
| Enteral feedings (mother's own milk) | -13.686 | 6.794 | -0.261 | -2.014 | 0.055 |
