# Supplementary material for: Dichotomous development of the gut microbiome in preterm infants
Source: Microbiome. 2018 Sep 12;6:157. doi: 10.1186/s40168-018-0547-8 (PMC6136210; doi:10.1186/s40168-018-0547-8)
Supplement: Supplementary file 3 — Unweighted UniFrac distance matrix for Fig. 4. (DOC 63 kb) [file 40168_2018_547_MOESM3_ESM.doc]

"# This is a submission template for batch deposit of 'MIMARKS: specimen, human-gut; version 4.0' samples to the NCBI BioSample database (http://www.ncbi.nlm.nih.gov/biosample/)."

"# GREEN fields are mandatory. Your submission will fail if any mandatory fields are not completed. If information is unavailable for any mandatory field, please enter 'not collected', 'not applicable' or 'missing' as appropriate."

"# BLUE fields indicate that at least one of those fields is mandatory. If information is unavailable, please enter 'not collected', 'not applicable' or 'missing' as appropriate."

# YELLOW fields are optional. Leave optional fields empty (or delete them) if no information is available.

"# You can add any number of custom fields to fully describe your BioSamples, simply include them in the table."

"# Hover over field name to view definition, or see http://www.ncbi.nlm.nih.gov/biosample/docs/attributes/."

"# CAUTION: Be aware that Excel may automatically apply formatting to your data. In particular, take care with dates, incrementing autofills and special characters like / or -. Doublecheck that your text file is accurate before uploading to BioSample."

# TO MAKE A SUBMISSION:

# 1. Complete this template table.

# 2. Upload the file on the 'Attributes' tab of the BioSample Submission Portal at https://submit.ncbi.nlm.nih.gov/subs/biosample/.

"# If you have any questions, please contact us at biosamplehelp@ncbi.nlm.nih.gov."

#

*sample_name sample_title bioproject_accession *organism strain isolate cultivar ecotype *collection_date *env_biome *env_feature *env_material *geo_loc_name *host *isol_growth_condt *lat_lon biotic_relationship chem_administration ethnicity extrachrom_elements gastrointest_disord host_age host_body_mass_index host_body_product host_body_temp host_diet host_disease host_family_relationship host_genotype host_height host_last_meal host_occupation host_phenotype host_pulse host_sex host_subject_id host_tissue_sampled host_tot_mass ihmc_medication_code isolation_source liver_disord medic_hist_perform vaginal_delivery proteo_cluster gestational_age_at_delivery organism_count oxy_stat_samp perturbation rel_to_oxygen samp_collect_device samp_mat_process samp_salinity samp_size samp_store_dur samp_store_loc samp_store_temp samp_vol_we_dna_ext source_material_id special_diet subspecf_gen_lin temp trophic_level description

Infant2_1 uncultured bacteria InfantInfant2_1 stool 6/17/12 human infant stool USA:Tampa Homo sapiens not applicable not collected Non-Hispanic 12 days stool Female 0 2 31.71

Infant2_2 uncultured bacteria InfantInfant2_2 stool 6/23/12 human infant stool USA:Tampa Homo sapiens not applicable not collected Non-Hispanic 18 days stool Female 0 2 31.71

Infant2_3 uncultured bacteria InfantInfant2_3 stool 7/3/12 human infant stool USA:Tampa Homo sapiens not applicable not collected Non-Hispanic 28 days stool Female 0 2 31.71

Infant4_1 uncultured bacteria InfantInfant4_1 stool 6/21/12 human infant stool USA:Tampa Homo sapiens not applicable not collected Hispanic 8 days stool Female 0 1 29.29

Infant4_2 uncultured bacteria InfantInfant4_2 stool 7/6/12 human infant stool USA:Tampa Homo sapiens not applicable not collected Hispanic 23 days stool Female 0 1 29.29

Infant4_3 uncultured bacteria InfantInfant4_3 stool 7/11/12 human infant stool USA:Tampa Homo sapiens not applicable not collected Hispanic 28 days stool Female 0 1 29.29

Infant6_1 uncultured bacteria InfantInfant6_1 stool 7/16/12 human infant stool USA:Tampa Homo sapiens not applicable not collected Non-Hispanic 12 days stool Male 0 1 27.14

Infant6_2 uncultured bacteria InfantInfant6_2 stool 7/30/12 human infant stool USA:Tampa Homo sapiens not applicable not collected Non-Hispanic 26 days stool Male 0 1 27.14

Infant6_3 uncultured bacteria InfantInfant6_3 stool 8/5/12 human infant stool USA:Tampa Homo sapiens not applicable not collected Non-Hispanic 32 days stool Male 0 1 27.14

Infant8_1 uncultured bacteria InfantInfant8_1 stool 7/22/13 human infant stool USA:Tampa Homo sapiens not applicable not collected Hispanic 13 days stool Female 0 1 27

Infant8_2 uncultured bacteria InfantInfant8_2 stool 7/30/12 human infant stool USA:Tampa Homo sapiens not applicable not collected Hispanic 21 days stool Female 0 1 27

Infant8_3 uncultured bacteria InfantInfant8_3 stool 8/5/12 human infant stool USA:Tampa Homo sapiens not applicable not collected Hispanic 27 days stool Female 0 1 27

Infant10_1 uncultured bacteria InfantInfant10_1 stool 7/26/12 human infant stool USA:Tampa Homo sapiens not applicable not collected Hispanic 11 days stool Male 0 2 29.71

Infant10_2 uncultured bacteria InfantInfant10_2 stool 8/1/12 human infant stool USA:Tampa Homo sapiens not applicable not collected Hispanic 17 days stool Male 0 2 29.71

Infant10_3 uncultured bacteria InfantInfant10_3 stool 8/14/12 human infant stool USA:Tampa Homo sapiens not applicable not collected Hispanic 30 days stool Male 0 2 29.71

Infant11_1 uncultured bacteria InfantInfant11_1 stool 9/9/12 human infant stool USA:Tampa Homo sapiens not applicable not collected Hispanic 12 days stool Male 0 2 26.43

Infant11_2 uncultured bacteria InfantInfant11_2 stool 9/17/12 human infant stool USA:Tampa Homo sapiens not applicable not collected Hispanic 20 days stool Male 0 2 26.43

Infant11_3 uncultured bacteria InfantInfant11_3 stool 10/1/12 human infant stool USA:Tampa Homo sapiens not applicable not collected Hispanic 34 days stool Male 0 2 26.43

Infant13_1 uncultured bacteria InfantInfant13_1 stool 9/13/12 human infant stool USA:Tampa Homo sapiens not applicable not collected Non-Hispanic 15 days stool Female 0 2 31.86

Infant13_2 uncultured bacteria InfantInfant13_2 stool 9/20/12 human infant stool USA:Tampa Homo sapiens not applicable not collected Non-Hispanic 22 days stool Female 0 2 31.86

Infant13_3 uncultured bacteria InfantInfant13_3 stool 9/23/12 human infant stool USA:Tampa Homo sapiens not applicable not collected Non-Hispanic 25 days stool Female 0 2 31.86

Infant15_1 uncultured bacteria InfantInfant15_1 stool 9/25/12 human infant stool USA:Tampa Homo sapiens not applicable not collected Hispanic 7 days stool Male 1 2 28.71

Infant15_2 uncultured bacteria InfantInfant15_2 stool 10/12/12 human infant stool USA:Tampa Homo sapiens not applicable not collected Hispanic 24 days stool Male 1 2 28.71

Infant15_3 uncultured bacteria InfantInfant15_3 stool 10/22/12 human infant stool USA:Tampa Homo sapiens not applicable not collected Hispanic 34 days stool Male 1 2 28.71

Infant18_1 uncultured bacteria InfantInfant18_1 stool 10/4/12 human infant stool USA:Tampa Homo sapiens not applicable not collected Non-Hispanic 9 days stool Male 0 2 28.57

Infant18_2 uncultured bacteria InfantInfant18_2 stool 10/13/12 human infant stool USA:Tampa Homo sapiens not applicable not collected Non-Hispanic 18 days stool Male 0 2 28.57

Infant18_3 uncultured bacteria InfantInfant18_3 stool 10/21/12 human infant stool USA:Tampa Homo sapiens not applicable not collected Non-Hispanic 26 days stool Male 0 2 28.57

Infant22_1 uncultured bacteria InfantInfant22_1 stool 10/21/12 human infant stool USA:Tampa Homo sapiens not applicable not collected Non-Hispanic 7 days stool Female 0 1 28.71

Infant22_2 uncultured bacteria InfantInfant22_2 stool 11/5/12 human infant stool USA:Tampa Homo sapiens not applicable not collected Non-Hispanic 22 days stool Female 0 1 28.71

Infant22_3 uncultured bacteria InfantInfant22_3 stool 11/17/12 human infant stool USA:Tampa Homo sapiens not applicable not collected Non-Hispanic 34 days stool Female 0 1 28.71

Infant24_1 uncultured bacteria InfantInfant24_1 stool 10/30/12 human infant stool USA:Tampa Homo sapiens not applicable not collected Non-Hispanic 10 days stool Female 0 2 29.71

Infant24_2 uncultured bacteria InfantInfant24_2 stool 11/14/12 human infant stool USA:Tampa Homo sapiens not applicable not collected Non-Hispanic 25 days stool Female 0 2 29.71

Infant24_3 uncultured bacteria InfantInfant24_3 stool 11/18/12 human infant stool USA:Tampa Homo sapiens not applicable not collected Non-Hispanic 29 days stool Female 0 2 29.71

Infant25_1 uncultured bacteria InfantInfant25_1 stool 10/27/12 human infant stool USA:Tampa Homo sapiens not applicable not collected Non-Hispanic 6 days stool Female 1 2 28

Infant25_2 uncultured bacteria InfantInfant25_2 stool 11/13/12 human infant stool USA:Tampa Homo sapiens not applicable not collected Non-Hispanic 23 days stool Female 1 2 28

Infant25_3 uncultured bacteria InfantInfant25_3 stool 11/17/12 human infant stool USA:Tampa Homo sapiens not applicable not collected Non-Hispanic 27 days stool Female 1 2 28

Infant26_1 uncultured bacteria InfantInfant26_1 stool 11/5/12 human infant stool USA:Tampa Homo sapiens not applicable not collected Non-Hispanic 13 days stool Female 1 2 26.71

Infant26_2 uncultured bacteria InfantInfant26_2 stool 11/12/12 human infant stool USA:Tampa Homo sapiens not applicable not collected Non-Hispanic 20 days stool Female 1 2 26.71

Infant26_3 uncultured bacteria InfantInfant26_3 stool 11/26/12 human infant stool USA:Tampa Homo sapiens not applicable not collected Non-Hispanic 34 days stool Female 1 2 26.71

Infant28_1 uncultured bacteria InfantInfant28_1 stool 11/18/12 human infant stool USA:Tampa Homo sapiens not applicable not collected Non-Hispanic NEC 11 days stool Male 0 1 26.29

Infant28_2 uncultured bacteria InfantInfant28_2 stool 11/26/12 human infant stool USA:Tampa Homo sapiens not applicable not collected Non-Hispanic NEC 19 days stool Male 0 1 26.29

Infant28_3 uncultured bacteria InfantInfant28_3 stool 12/10/12 human infant stool USA:Tampa Homo sapiens not applicable not collected Non-Hispanic NEC 33 days stool Male 0 1 26.29

Infant29_1 uncultured bacteria InfantInfant29_1 stool 12/3/12 human infant stool USA:Tampa Homo sapiens not applicable not collected Non-Hispanic 15 days stool Male 0 2 28

Infant29_2 uncultured bacteria InfantInfant29_2 stool 12/10/12 human infant stool USA:Tampa Homo sapiens not applicable not collected Non-Hispanic 22 days stool Male 0 2 28

Infant29_3 uncultured bacteria InfantInfant29_3 stool 12/16/12 human infant stool USA:Tampa Homo sapiens not applicable not collected Non-Hispanic 28 days stool Male 0 2 28

Infant30_2 uncultured bacteria InfantInfant30_2 stool 12/5/12 human infant stool USA:Tampa Homo sapiens not applicable not collected Non-Hispanic 17 days stool Male 0 28.71

Infant30_3 uncultured bacteria InfantInfant30_3 stool 12/20/12 human infant stool USA:Tampa Homo sapiens not applicable not collected Non-Hispanic 32 days stool Male 0 28.71

Infant32_1 uncultured bacteria InfantInfant32_1 stool 12/31/12 human infant stool USA:Tampa Homo sapiens not applicable not collected Non-Hispanic 8 days stool Female 1 2 25

Infant32_3 uncultured bacteria InfantInfant32_3 stool 1/20/13 human infant stool USA:Tampa Homo sapiens not applicable not collected Non-Hispanic 28 days stool Female 1 2 25

Infant33_1 uncultured bacteria InfantInfant33_1 stool 12/27/12 human infant stool USA:Tampa Homo sapiens not applicable not collected Hispanic 2 days stool Female 1 2 26.43

Infant33_2 uncultured bacteria InfantInfant33_2 stool 1/16/13 human infant stool USA:Tampa Homo sapiens not applicable not collected Hispanic 22 days stool Female 1 2 26.43

Infant33_3 uncultured bacteria InfantInfant33_3 stool 1/22/13 human infant stool USA:Tampa Homo sapiens not applicable not collected Hispanic 28 days stool Female 1 2 26.43

Infant34_1 uncultured bacteria InfantInfant34_1 stool 12/31/12 human infant stool USA:Tampa Homo sapiens not applicable not collected Non-Hispanic 7 days stool Female 0 1 30.71

Infant34_2 uncultured bacteria InfantInfant34_2 stool 1/12/13 human infant stool USA:Tampa Homo sapiens not applicable not collected Non-Hispanic 19 days stool Female 0 1 30.71

Infant34_3 uncultured bacteria InfantInfant34_3 stool 1/26/13 human infant stool USA:Tampa Homo sapiens not applicable not collected Non-Hispanic 33 days stool Female 0 1 30.71

Infant36_1 uncultured bacteria InfantInfant36_1 stool 1/23/13 human infant stool USA:Tampa Homo sapiens not applicable not collected Hispanic 14 days stool Male 0 2 25.57

Infant36_2 uncultured bacteria InfantInfant36_2 stool 1/30/13 human infant stool USA:Tampa Homo sapiens not applicable not collected Hispanic 21 days stool Male 0 2 25.57

Infant36_3 uncultured bacteria InfantInfant36_3 stool 2/6/13 human infant stool USA:Tampa Homo sapiens not applicable not collected Hispanic 28 days stool Male 0 2 25.57

Infant37_1 uncultured bacteria InfantInfant37_1 stool 1/27/13 human infant stool USA:Tampa Homo sapiens not applicable not collected Non-Hispanic 7 days stool Male 1 1 28.14

Infant37_2 uncultured bacteria InfantInfant37_2 stool 2/10/13 human infant stool USA:Tampa Homo sapiens not applicable not collected Non-Hispanic 21 days stool Male 1 1 28.14

Infant37_3 uncultured bacteria InfantInfant37_3 stool 2/17/13 human infant stool USA:Tampa Homo sapiens not applicable not collected Non-Hispanic 25 days stool Male 1 1 28.14

Infant38_1 uncultured bacteria InfantInfant38_1 stool 2/2/13 human infant stool USA:Tampa Homo sapiens not applicable not collected Non-Hispanic 10 days stool Female 1 2 28.71

Infant38_2 uncultured bacteria InfantInfant38_2 stool 2/14/13 human infant stool USA:Tampa Homo sapiens not applicable not collected Non-Hispanic 22 days stool Female 1 2 28.71

Infant38_3 uncultured bacteria InfantInfant38_3 stool 2/20/13 human infant stool USA:Tampa Homo sapiens not applicable not collected Non-Hispanic 28 days stool Female 1 2 28.71

Infant41_1 uncultured bacteria InfantInfant41_1 stool 2/5/13 human infant stool USA:Tampa Homo sapiens not applicable not collected Non-Hispanic 7 days stool Male 1 2 32

Infant41_2 uncultured bacteria InfantInfant41_2 stool 2/12/13 human infant stool USA:Tampa Homo sapiens not applicable not collected Non-Hispanic 14 days stool Male 1 2 32

Infant41_3 uncultured bacteria InfantInfant41_3 stool 2/18/13 human infant stool USA:Tampa Homo sapiens not applicable not collected Non-Hispanic 20 days stool Male 1 2 32

Infant44_1 uncultured bacteria InfantInfant44_1 stool 3/1/13 human infant stool USA:Tampa Homo sapiens not applicable not collected Non-Hispanic 6 days stool Female 0 1 27.43

Infant44_2 uncultured bacteria InfantInfant44_2 stool 3/21/13 human infant stool USA:Tampa Homo sapiens not applicable not collected Non-Hispanic 26 days stool Female 0 1 27.43

Infant44_3 uncultured bacteria InfantInfant44_3 stool 3/28/13 human infant stool USA:Tampa Homo sapiens not applicable not collected Non-Hispanic 33 days stool Female 0 1 27.43

Infant45_1 uncultured bacteria InfantInfant45_1 stool 3/11/13 human infant stool USA:Tampa Homo sapiens not applicable not collected Non-Hispanic 14 days stool Male 0 1 27.29

Infant45_2 uncultured bacteria InfantInfant45_2 stool 3/15/13 human infant stool USA:Tampa Homo sapiens not applicable not collected Non-Hispanic 18 days stool Male 0 1 27.29

Infant45_3 uncultured bacteria InfantInfant45_3 stool 3/25/13 human infant stool USA:Tampa Homo sapiens not applicable not collected Non-Hispanic 28 days stool Male 0 1 27.29

Infant46_1 uncultured bacteria InfantInfant46_1 stool 3/25/13 human infant stool USA:Tampa Homo sapiens not applicable not collected Non-Hispanic 10 days stool Female 0 2 27.29

Infant46_2 uncultured bacteria InfantInfant46_2 stool 4/8/13 human infant stool USA:Tampa Homo sapiens not applicable not collected Non-Hispanic 24 days stool Female 0 2 27.29

Infant46_3 uncultured bacteria InfantInfant46_3 stool 4/13/13 human infant stool USA:Tampa Homo sapiens not applicable not collected Non-Hispanic 29 days stool Female 0 2 27.29

Infant47_1 uncultured bacteria InfantInfant47_1 stool 4/10/13 human infant stool USA:Tampa Homo sapiens not applicable not collected Non-Hispanic 10 days stool Female 0 1 29.43

Infant47_2 uncultured bacteria InfantInfant47_2 stool 4/25/13 human infant stool USA:Tampa Homo sapiens not applicable not collected Non-Hispanic 25 days stool Female 0 1 29.43

Infant47_3 uncultured bacteria InfantInfant47_3 stool 5/2/13 human infant stool USA:Tampa Homo sapiens not applicable not collected Non-Hispanic 32 days stool Female 0 1 29.43

Infant50_1 uncultured bacteria InfantInfant50_1 stool 4/10/13 human infant stool USA:Tampa Homo sapiens not applicable not collected Non-Hispanic 6 days stool Female 0 1 28.57

Infant50_2 uncultured bacteria InfantInfant50_2 stool 4/25/13 human infant stool USA:Tampa Homo sapiens not applicable not collected Non-Hispanic 21 days stool Female 0 1 28.57

Infant50_3 uncultured bacteria InfantInfant50_3 stool 5/2/13 human infant stool USA:Tampa Homo sapiens not applicable not collected Non-Hispanic 28 days stool Female 0 1 28.57

Infant51_1 uncultured bacteria InfantInfant51_1 stool 4/26/13 human infant stool USA:Tampa Homo sapiens not applicable not collected Non-Hispanic 7 days stool Male 0 1 24

Infant51_2 uncultured bacteria InfantInfant51_2 stool 5/12/13 human infant stool USA:Tampa Homo sapiens not applicable not collected Non-Hispanic 23 days stool Male 0 1 24

Infant51_3 uncultured bacteria InfantInfant51_3 stool 5/20/13 human infant stool USA:Tampa Homo sapiens not applicable not collected Non-Hispanic 31 days stool Male 0 1 24

Infant52_1 uncultured bacteria InfantInfant52_1 stool 4/25/13 human infant stool USA:Tampa Homo sapiens not applicable not collected Non-Hispanic 8 days stool Female 0 1 29.71

Infant52_2 uncultured bacteria InfantInfant52_2 stool 5/9/13 human infant stool USA:Tampa Homo sapiens not applicable not collected Non-Hispanic 22 days stool Female 0 1 29.71

Infant52_3 uncultured bacteria InfantInfant52_3 stool 5/15/13 human infant stool USA:Tampa Homo sapiens not applicable not collected Non-Hispanic 28 days stool Female 0 1 29.71

Infant53_1 uncultured bacteria InfantInfant53_1 stool 4/25/13 human infant stool USA:Tampa Homo sapiens not applicable not collected Non-Hispanic 7 days stool Male 0 1 29.71

Infant53_2 uncultured bacteria InfantInfant53_2 stool 5/14/13 human infant stool USA:Tampa Homo sapiens not applicable not collected Non-Hispanic 26 days stool Male 0 1 29.71

Infant53_3 uncultured bacteria InfantInfant53_3 stool 5/23/13 human infant stool USA:Tampa Homo sapiens not applicable not collected Non-Hispanic 35 days stool Male 0 1 29.71

Infant56_1 uncultured bacteria InfantInfant56_1 stool 5/14/13 human infant stool USA:Tampa Homo sapiens not applicable not collected Non-Hispanic 13 days stool Female 0 1 29

Infant56_2 uncultured bacteria InfantInfant56_2 stool 5/20/13 human infant stool USA:Tampa Homo sapiens not applicable not collected Non-Hispanic 19 days stool Female 0 1 29

Infant56_3 uncultured bacteria InfantInfant56_3 stool 6/3/13 human infant stool USA:Tampa Homo sapiens not applicable not collected Non-Hispanic 32 days stool Female 0 1 29

Infant57_1 uncultured bacteria InfantInfant57_1 stool 5/26/13 human infant stool USA:Tampa Homo sapiens not applicable not collected Non-Hispanic 13 days stool Female 1 2 30.14

Infant57_2 uncultured bacteria InfantInfant57_2 stool 6/3/13 human infant stool USA:Tampa Homo sapiens not applicable not collected Non-Hispanic 21 days stool Female 1 2 30.14

Infant57_3 uncultured bacteria InfantInfant57_3 stool 6/16/13 human infant stool USA:Tampa Homo sapiens not applicable not collected Non-Hispanic 34 days stool Female 0 2 30.14

Infant61_1 uncultured bacteria InfantInfant61_1 stool 6/17/13 human infant stool USA:Tampa Homo sapiens not applicable not collected Non-Hispanic 10 days stool Male 1 2 26.29

Infant61_2 uncultured bacteria InfantInfant61_2 stool 7/1/13 human infant stool USA:Tampa Homo sapiens not applicable not collected Non-Hispanic 24 days stool Male 1 2 26.29

Infant61_3 uncultured bacteria InfantInfant61_3 stool 7/13/13 human infant stool USA:Tampa Homo sapiens not applicable not collected Non-Hispanic 36 days stool Male 1 2 26.29

Infant64_1 uncultured bacteria InfantInfant64_1 stool 7/2/13 human infant stool USA:Tampa Homo sapiens not applicable not collected Non-Hispanic 13 days stool Female 1 2 30.86

Infant64_2 uncultured bacteria InfantInfant64_2 stool 7/14/13 human infant stool USA:Tampa Homo sapiens not applicable not collected Non-Hispanic 25 days stool Female 1 2 30.86

Infant64_3 uncultured bacteria InfantInfant64_3 stool 7/18/13 human infant stool USA:Tampa Homo sapiens not applicable not collected Non-Hispanic 29 days stool Female 1 2 30.86

Infant69_1 uncultured bacteria InfantInfant69_1 stool 9/2/13 human infant stool USA:Tampa Homo sapiens not applicable not collected Hispanic 10 days stool Female 0 2 29.14

Infant69_2 uncultured bacteria InfantInfant69_2 stool 9/9/13 human infant stool USA:Tampa Homo sapiens not applicable not collected Hispanic 17 days stool Female 0 2 29.14

Infant69_3 uncultured bacteria InfantInfant69_3 stool 9/22/13 human infant stool USA:Tampa Homo sapiens not applicable not collected Hispanic 30 days stool Female 0 2 29.14

Infant70_1 uncultured bacteria InfantInfant70_1 stool 9/9/13 human infant stool USA:Tampa Homo sapiens not applicable not collected Non-Hispanic 13 days stool Male 0 1 28.43

Infant70_2 uncultured bacteria InfantInfant70_2 stool 9/16/13 human infant stool USA:Tampa Homo sapiens not applicable not collected Non-Hispanic 20 days stool Male 0 1 28.43

Infant70_3 uncultured bacteria InfantInfant70_3 stool 9/29/13 human infant stool USA:Tampa Homo sapiens not applicable not collected Non-Hispanic 33 days stool Male 0 1 28.43

Infant71_1 uncultured bacteria InfantInfant71_1 stool 9/5/13 human infant stool USA:Tampa Homo sapiens not applicable not collected Non-Hispanic 12 days stool Male 0 2 27.71

Infant71_2 uncultured bacteria InfantInfant71_2 stool 9/19/13 human infant stool USA:Tampa Homo sapiens not applicable not collected Non-Hispanic 26 days stool Male 0 2 27.71

Infant71_3 uncultured bacteria InfantInfant71_3 stool 9/24/13 human infant stool USA:Tampa Homo sapiens not applicable not collected Non-Hispanic 31 days stool Male 0 2 27.71

Infant72_1 uncultured bacteria InfantInfant72_1 stool 9/29/13 human infant stool USA:Tampa Homo sapiens not applicable not collected Non-Hispanic 11 days stool Female 0 2 26.57

Infant72_2 uncultured bacteria InfantInfant72_2 stool 10/14/13 human infant stool USA:Tampa Homo sapiens not applicable not collected Non-Hispanic 26 days stool Female 0 2 26.57

Infant72_3 uncultured bacteria InfantInfant72_3 stool 10/21/13 human infant stool USA:Tampa Homo sapiens not applicable not collected Non-Hispanic 33 days stool Female 0 2 26.57

Infant73_1 uncultured bacteria InfantInfant73_1 stool 9/29/13 human infant stool USA:Tampa Homo sapiens not applicable not collected Non-Hispanic 11 days stool Female 0 1 26.57

Infant73_2 uncultured bacteria InfantInfant73_2 stool 10/14/13 human infant stool USA:Tampa Homo sapiens not applicable not collected Non-Hispanic 26 days stool Female 0 1 26.57

Infant73_3 uncultured bacteria InfantInfant73_3 stool 10/20/13 human infant stool USA:Tampa Homo sapiens not applicable not collected Non-Hispanic 32 days stool Female 0 1 26.57

Infant74_1 uncultured bacteria InfantInfant74_1 stool 10/25/13 human infant stool USA:Tampa Homo sapiens not applicable not collected Non-Hispanic 13 days stool Male 0 2 27.86

Infant74_2 uncultured bacteria InfantInfant74_2 stool 10/31/13 human infant stool USA:Tampa Homo sapiens not applicable not collected Non-Hispanic 19 days stool Male 0 2 27.86

Infant74_3 uncultured bacteria InfantInfant74_3 stool 11/7/13 human infant stool USA:Tampa Homo sapiens not applicable not collected Non-Hispanic 26 days stool Male 0 2 27.86

Infant78_1 uncultured bacteria InfantInfant78_1 stool 12/6/13 human infant stool USA:Tampa Homo sapiens not applicable not collected Hispanic 14 days stool Male 0 1 26.71

Infant78_2 uncultured bacteria InfantInfant78_2 stool 12/12/13 human infant stool USA:Tampa Homo sapiens not applicable not collected Hispanic 20 days stool Male 0 1 26.71

Infant78_3 uncultured bacteria InfantInfant78_3 stool 12/19/13 human infant stool USA:Tampa Homo sapiens not applicable not collected Hispanic 27 days stool Male 0 1 26.71

Infant79_1 uncultured bacteria InfantInfant79_1 stool 12/1/13 human infant stool USA:Tampa Homo sapiens not applicable not collected Non-Hispanic 10 days stool Male 0 2 27.86

Infant79_2 uncultured bacteria InfantInfant79_2 stool 12/9/13 human infant stool USA:Tampa Homo sapiens not applicable not collected Non-Hispanic 18 days stool Male 0 2 27.86

Infant79_3 uncultured bacteria InfantInfant79_3 stool 12/22/13 human infant stool USA:Tampa Homo sapiens not applicable not collected Non-Hispanic 31 days stool Male 0 2 27.86

Infant81_1 uncultured bacteria InfantInfant81_1 stool 12/15/13 human infant stool USA:Tampa Homo sapiens not applicable not collected Non-Hispanic 6 days stool Female 0 1 26.71

Infant81_2 uncultured bacteria InfantInfant81_2 stool 12/30/13 human infant stool USA:Tampa Homo sapiens not applicable not collected Non-Hispanic 21 days stool Female 0 1 26.71

Infant81_3 uncultured bacteria InfantInfant81_3 stool 1/6/14 human infant stool USA:Tampa Homo sapiens not applicable not collected Non-Hispanic 28 days stool Female 0 1 26.71

Infant82_1 uncultured bacteria InfantInfant82_1 stool 12/13/13 human infant stool USA:Tampa Homo sapiens not applicable not collected Non-Hispanic 8 days stool Male 0 1 37

Infant82_3 uncultured bacteria InfantInfant82_3 stool 12/30/13 human infant stool USA:Tampa Homo sapiens not applicable not collected Non-Hispanic 24 days stool Male 0 1 37
